# Anoctamin-5 deficiency enhances ATG9A-dependent autophagy, inducing osteogenesis and gnathodiaphyseal dysplasia–like bone formation

**DOI:** 10.1172/jci.insight.189817

**Published:** 2025-03-11

**Authors:** Shuai Zhang, Shengnan Wang, Sirui Liu, Xiu Liu, Mingyue Zhang, Huichong Xu, Xiaoyu Wang, Hongyu Li, Ying Hu

**Affiliations:** Beijing Institute of Dental Research, Beijing Stomatological Hospital, Capital Medical University, Beijing, China.

**Keywords:** Bone biology, Genetics, Autophagy, Bone disease

## Abstract

Mutations in the anoctamin-5 (*ANO5*) gene can lead to musculoskeletal disorders, with monoallelic (autosomal dominant) mutations typically presenting as skeletal abnormalities known as gnathodiaphyseal dysplasia (GDD). Clinically, GDD is characterized by thickened cortices of long bones and mandibles, narrowed medullary cavities, and increased bone fragility. While autophagy is necessary in regulating bone formation, the specific relationship between *ANO5* and autophagy remains poorly understood. In this study, we demonstrated that *Ano5* deficiency activates autophagy in mouse cranial osteoblasts (mCOBs), leading to enhanced osteogenic capacity in *Ano5^–/–^* mCOBs. The application of 3-methyladenine (3-MA) and chloroquine (CQ) reversed the excessive osteogenesis observed in *Ano5^–/–^* mCOBs. Further analysis revealed that *Ano5* deficiency upregulated the expression of ATG9A, and silencing ATG9A significantly reduced both autophagy and osteogenic activity in *Ano5^–/–^* mCOBs. Additionally, AMP-activated protein kinase (AMPK) was found to positively regulate ATG9A, and inhibiting AMPK reduced ATG9A expression, which in turn mitigated excessive osteogenesis of *Ano5^–/–^* mCOBs. Moreover, in vivo experiments confirmed that treatment with 3-MA alleviated the bone phenotype abnormalities in *Ano5^–/–^* mice. These findings suggest that *Ano5* negatively regulates autophagy, contributing to illuminate pathogenesis of GDD. Meanwhile, this research highlights potential therapeutic strategies targeting autophagy to pave the way for the clinical manifestations of GDD.

## Abstract 2

The role and function of anoctamin 5 in gnathodiaphyseal dysplasia

## Graphical abstract

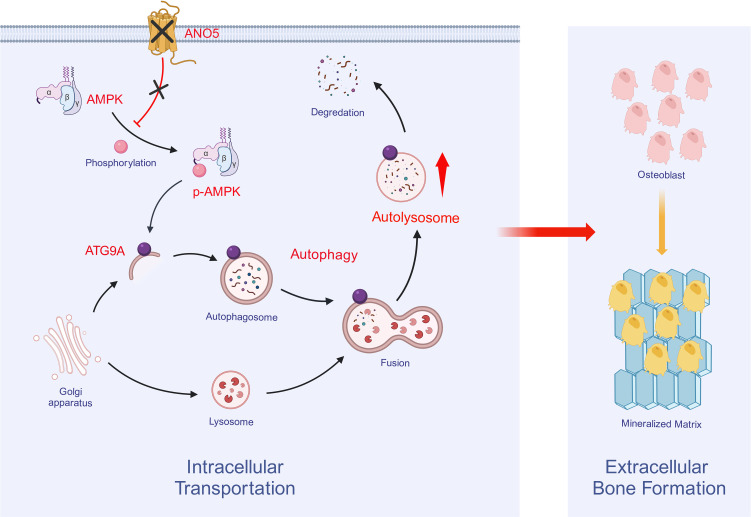

## Introduction

Anoctamin-5 (ANO5), which is encoded by the *ANO5* gene and a member of the TMEM16 transmembrane protein family, is highly expressed in skeletal muscle and bone tissues. Several members of this protein family have been shown to function as calcium-activated chloride channels. However, structural predictions and ion channel assays suggest that ANO5 exhibits minimal ion channel activity (1, 2). Despite this, ANO5 has a significant correlation with intracellular calcium oscillations (3). Mutations in the *ANO5* gene are associated with muscular or skeletal diseases, with recessive mutations manifesting as muscular disorders, commonly described as limb-girdle muscular dystrophy R12 and Miyoshi muscular dystrophy type 3, and dominant mutations presenting as skeletal disorders, notably gnathodiaphyseal dysplasia (GDD) (4).

GDD (OMIM #166260) is a rare autosomal dominant skeletal disorder characterized by craniofacial deformities and abnormalities in both the femurs and tibiae. In the craniofacial region, patients typically present with abnormal jaw hypertrophy, facial disfigurement, and dental malocclusion or agenesis (5, 6). In the femurs and tibiae, GDD is associated with increased bone mineral density, narrowing of the medullary canal, and increased bone fragility, typically manifesting in childhood (7). Radiographic imaging shows irregular radiopaque lesions resembling a “cotton wool” pattern around the tooth roots, with bowing and thickening of long bones, particularly in the tibiae (3, 8). Some of these features are similar to those of osteopetrosis (9).

The most commonly affected sites in patients with GDD are cysteine residues at positions 356 (c.1067 G) and 360 (c.1079 G) in exons 11–12 (4). Therefore, our group previously established a knockout mouse model in which *Ano5* exons 11 and 12 were deleted, which successfully exhibited bone remodeling defects reminiscent of GDD and represented an appropriate model to explore the GDD mechanism. Moreover, osteoblasts derived from these mice showed enhanced osteogenic activity and increased expression of osteogenic markers in vitro (10). Although the mouse model does not fully replicate human GDD, its high protein conservation and the demonstrated potential for skeletal changes suggest that this model holds significant value. Currently, emerging evidence suggests that ANO5 may influence additional cellular processes. A recent clustering analysis of ANO5 protein interactions has revealed that ANO5 interacts with proteins related to protein degradation and vesicle transport (11). Furthermore, the loss of ANO5 has been associated with the accumulation of annexin A2, which has been reported to stimulate autophagy, indicating that ANO5 may play a broader role in cellular homeostasis (12, 13).

Autophagy, a lysosome-dependent degradation process, is a critical mechanism for maintaining cellular homeostasis and supporting bone remodeling. This process involves several key steps, including the formation, expansion, and maturation of autophagosomes, followed by their fusion with lysosomes to form autolysosomes. Autophagic vesicles, including autophagosomes and autolysosomes, also function as carriers for the secretion of hydroxyapatite by osteoblasts, directly contributing to the mineralization of the extracellular matrix (14). Dysregulation of autophagy has been implicated in several skeletal diseases, such as osteoarthritis, osteoporosis, and osteosclerosis. Given the significant role of autophagy in bone remodeling, its potential involvement in GDD presents a novel area of investigation.

The progression of autophagy relies on the coordination of various autophagy-related (ATG) proteins. Among these, ATG9A is the only multispanning transmembrane protein and plays a crucial role in the initial stages of autophagy. Structural studies have shown that ATG9A contains a network of internal cavities, facilitating the translocation of phospholipids across membranes. This function is critical for the expansion of the phagophore membrane, an essential step in autophagic vesicle formation. ATG9A is primarily localized to post-Golgi compartments, including the *trans*-Golgi network (TGN), and is one of the earliest proteins recruited to nascent autophagic vesicles. Notably, Ashari et al. found that ATG9A is regulated by AMP-activated protein kinase (AMPK) at the sites of autophagosome biogenesis (15). AMPK is a central regulator of cellular energy balance. In bone metabolism, AMPK activation has been implicated in osteoblast differentiation and bone remodeling by regulating energy homeostasis and autophagy-related pathways (16).

Given the clinical significance of ANO5 and the growing evidence of its relationship with autophagy, we have formally explored its role in regulating bone remodeling. Our findings suggest that ANO5 negatively regulates autophagy by modulating the AMPK/ATG9A pathway. This interaction not only provides what we believe are new insights into the molecular mechanisms of ANO5 in bone formation, but also provides preliminary insights into the pathogenesis of GDD and its potential therapeutic targets.

## Results

### Autophagic flux is promoted by the absence of Ano5 in mCOBs.

**Figure 1:**
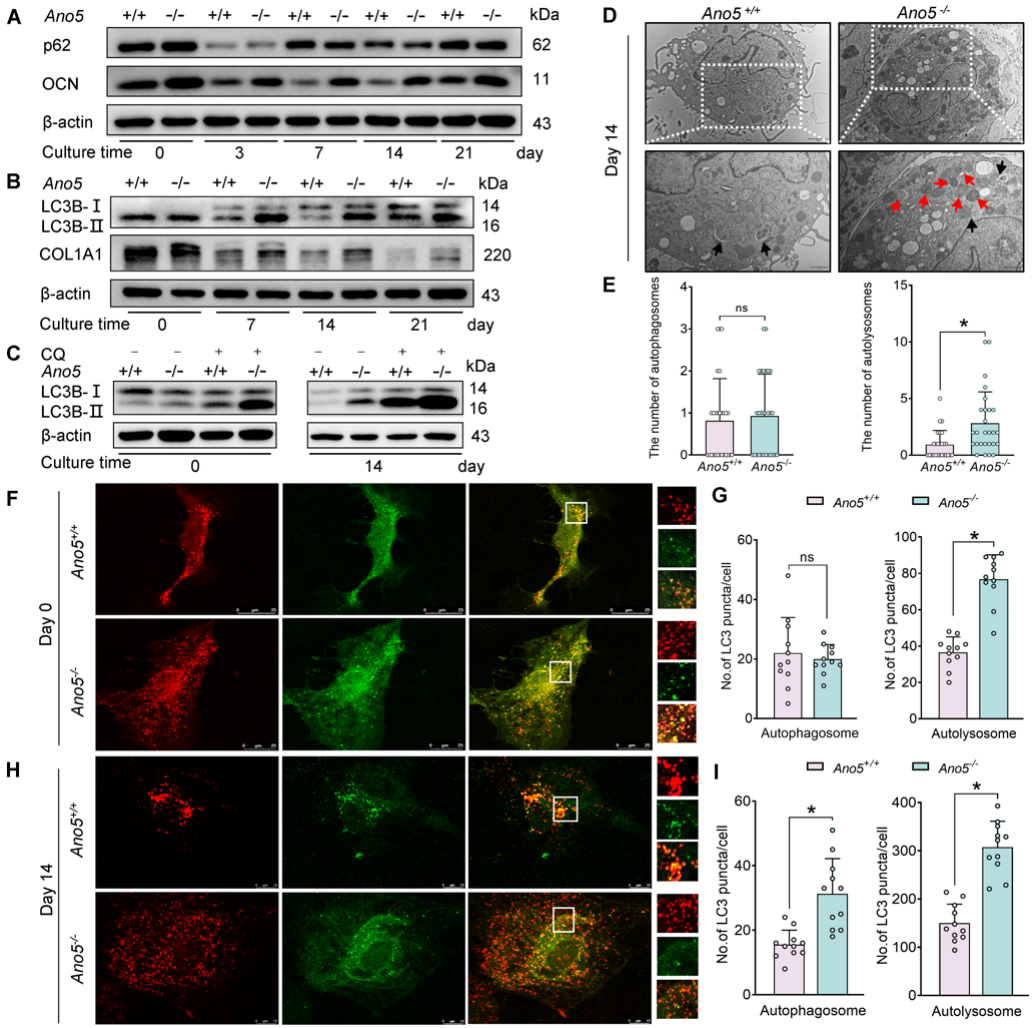
Autophagic flux is promoted by the absence of *Ano5* in mCOBs. (**A** and **B**) Western blot analysis of p62, OCN, LC3B-II, and COL1A1 protein levels in mCOBs from the *Ano5*^+/+^ and *Ano5^–/–^* groups during osteogenic differentiation. (**C**) Western blot showing the accumulation of LC3B-II in mCOBs from the *Ano5*^+/+^ and *Ano5^–/–^* groups after CQ treatment. (**D**) TEM images of mCOBs following 14 days of osteogenic induction, displaying autophagosomes (black arrowheads) and autolysosomes (red arrowheads). Scale bars: 2 μm (top) and 1 μm (bottom). (**E**) Quantitation of autophagosomes and autolysosomes observed in TEM images. (**F** and **H**) Confocal microscopy of mCOBs infected with mCherry-GFP-LC3B adenovirus, assessed on day 0 and day 14 of osteogenic induction. Representative images show autophagic activity. Scale bars: 25 μm (top rows) and 10 μm (bottom rows). The zoomed-in images in **F** are magnified ×800, and those in **H** are magnified ×1200. (**G** and **I**) Quantification of autophagosomes (mCherry^+^GFP^+^, yellow puncta) and autolysosomes (mCherry^+^GFP^–^, red puncta) in **F** and **H**, respectively. Data are represented as mean ± SD. *P < 0.05; NS, *P* > 0.05, as assessed by 1-way ANOVA followed by Tukey’s post hoc test.

To investigate whether autophagic activity is implicated in the absence of *Ano5*, we designed a series of experiments to explore the difference in autophagic activity between *Ano5^+/+^* and *Ano5^–/–^* mouse cranial osteoblasts (mCOBs). Data from experiments confirmed that the absence of *Ano5* caused a significant promotion of autophagic vesicle formation. As shown in Figure 1, A and B, the expression of osteocalcin (OCN) and collagen type I α1 (COL1A1) was notably elevated in *Ano5^–/–^* mCOBs compared with *Ano5^+/+^* mCOBs during osteogenic induction. Additionally, the autophagy marker LC3B-II exhibited a marked increase in *Ano5^–/–^* mCOBs relative to *Ano5^+/+^* mCOBs (Figure 1B), which is consistent with the results of Gene Ontology (GO) analysis based on RNA sequencing (RNA-Seq). The GO analysis revealed that autophagic activity was substantially different in mCOBs in the *Ano5^–/–^* group compared with those in the *Ano5^+/+^* group (Supplemental Figure 2; supplemental material available online with this article; https://doi.org/10.1172/jci.insight.189817DS1). However, the expression of another autophagy marker, p62, did not show obvious differences between these 2 groups (Figure 1A). Then, we conducted further experiments to assess autophagic flux to distinguish between increased autophagic vesicle production and impaired autophagic vesicle degradation, both of which may contribute to the accumulation of LC3B-II. Firstly, chloroquine (CQ) was utilized to inhibit the degradation of LC3B-II. The results indicated a greater accumulation of LC3B-II in *Ano5^–/–^* mCOBs, suggesting that the production of autophagic vesicles was enhanced (Figure 1C). Secondly, the results from transmission electron microscopy (TEM) showed that after osteogenic induction, the total amount of autolysosomes in *Ano5^–/–^* mCOBs exceeded that in *Ano5^+/+^* mCOBs, while the total amount of autophagosomes did not differ statistically between the 2 groups (Figure 1, D and E). Thirdly, the maturation of autophagic vesicles was monitored with autophagosomes labeled as yellow puncta and autolysosomes labeled as red puncta by using adenovirus expressing an mCherry-GFP-LC3B fusion protein. The results demonstrated that, prior to osteogenic induction, there was no significant change in the number of autophagosomes in *Ano5^–/–^* mCOBs compared to *Ano5^+/+^* mCOBs, whereas the number of autolysosomes increased significantly (Figure 1, F and G). Following osteogenic induction, both autophagosomes and autolysosomes in *Ano5^–/–^* mCOBs showed a marked increase, with the change in autolysosomes being particularly pronounced (Figure 1, H and I).

Additionally, Lyso-Tracker was utilized to assess the number of lysosomes, which is vital for autolysosome formation and degradation. We found that the number of lysosomes was substantially higher in *Ano5^–/–^* mCOBs compared with *Ano5^+/+^* mCOBs during osteogenic induction (Supplemental Figure 3A). Furthermore, the genes related to the integrity of lysosomes and the transportation of autophagosomes to lysosomes, including RAB7, member RAS oncogene family (*Rab7a*), lysosomal-associated membrane protein 1 (*Lamp1*), and vesicle-associated membrane protein 8 (*Vamp8*) were analyzed. The results showed that the expression of these genes in *Ano5^–/–^* mCOBs increased significantly compared with *Ano5^+/+^* mCOBs (Supplemental Figure 3B). These findings further corroborate the observation that *Ano5* deficiency led to notably increased autolysosomes in *Ano5^–/–^* mCOBs.

Subsequently, we examined relevant indicators in bone tissues. Notably, the thickness of the cartilage layer at the tibial epiphysis was significantly increased (Supplemental Figure 4, A and B), and OCN expression was elevated in the alveolar bone lacunae of the mandible in *Ano5^–/–^* mice (Supplemental Figure 4, C and D), indicating aberrant bone metabolic activity. Additionally, LC3B-II expression levels were markedly upregulated in various bone tissues, including the mandibles, tibiae, and femurs (Supplemental Figure 4, E and F). These findings suggest the presence of abnormal bone metabolism and autophagic activity within the bone tissues of the *Ano5*-deficient mice. Notably, both in mCOBs and bone tissues from *Ano5^–/–^* mice, autophagy and osteogenesis markers were significantly upregulated. This suggests a potential link between autophagy dysregulation and the abnormal osteogenic activity observed in this model.

### Inhibition of autophagy mitigates the aberrant osteogenic capacity in Ano5^–/–^ mCOBs.

**Figure 2:**
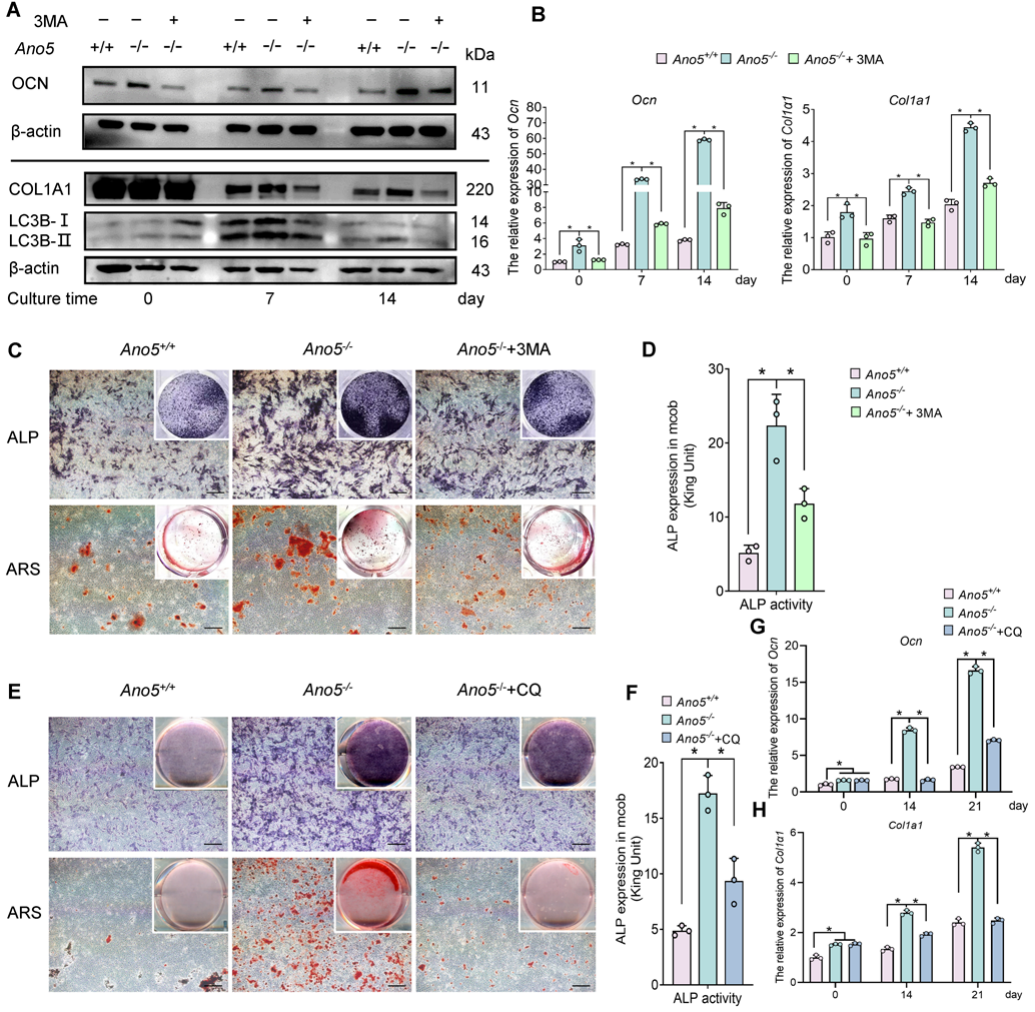
Inhibition of autophagy mitigates the aberrant osteogenic capacity in *Ano5^–/–^* mCOBs. 3-MA treatment: (**A**–**D**) mCOBs from the *Ano5^–/–^* group were treated with 3-MA (early-stage autophagy inhibitor) during osteogenic induction. (**A**) Western blot analysis of LC3B-II, OCN, and COL1A1 protein levels. (**B**) qRT-PCR analysis of *Ocn* and *Col1a1* expression. (**C**) ALP staining and alizarin red staining. Scale bars: 200 μm. (**D**) ALP activity assay. CQ treatment: (**E**–**H**) mCOBs were treated with CQ (late-stage autophagy inhibitor). (**E**) ALP staining and alizarin red staining. Scale bars: 200 μm. (**F**) ALP activity assay. (**G** and **H**) qRT-PCR analysis of *Ocn* and *Col1a1* expression. Data are represented as mean ± SD. *P < 0.05; NS, *P* > 0.05, as assessed by 1-way ANOVA followed by Tukey’s post hoc test.

To further examine whether the change in autophagic activity is associated with excessive osteogenesis in *Ano5^–/–^* mCOBs, 3-methyladenine (3-MA), which inhibits the formation of autophagic vesicles, was utilized in *Ano5^–/–^* mCOBs. The results showed that LC3B-II levels in *Ano5^–/–^* mCOBs was reduced following treatment with 3-MA after osteogenic induction (Figure 2A), accompanied by decreased protein and mRNA levels of OCN and COL1A1 after osteogenic induction (Figure 2, A and B). We then assessed the degree of osteogenic differentiation in the cells. Results showed that the alkaline phosphatase (ALP) activity, which represents the early stage of osteogenic differentiation, was higher in *Ano5^–/–^* mCOBs compared with *Ano5^+/+^* mCOBs, and it was significantly reduced following treatment with 3-MA (Figure 2, C and D). Additionally, calcium nodule formation, which signifies the late stage of osteogenic differentiation, was also substantially decreased in the 3-MA–treated group, as indicated by alizarin red staining (ARS) (Figure 2C). These results demonstrate that inhibiting the formation of autophagic vesicles significantly impairs the osteogenic potential of *Ano5^–/–^* mCOBs.

Consequently, we explored how inhibiting the degradation of autophagic vesicles affects osteogenesis, given that accumulated autophagic vesicles carrying minerals can enhance osteogenic capacity by being secreted into the extracellular matrix directly (17). Therefore, CQ was utilized to disrupt the function of lysosomes, thereby inhibiting the degradation of autophagic vesicles, and the results showed that the osteogenic capacity of the *Ano5^–/–^* mCOBs was also significantly reduced following the treatment with CQ, including reduced ALP activity and calcium nodules formation (Figure 2, E and F). Furthermore, the mRNA levels of *Ocn* and *Col1a1* were markedly downregulated in the CQ-treated group (Figure 2, G and H). These findings suggest that either inhibiting the formation or degradation of autophagic vesicles in *Ano5^–/–^* mCOBs could alleviate their abnormal osteogenic capacity, indicating a close correlation between the change in autophagy progression and the excessive osteogenesis in *Ano5^–/–^* mCOBs.

### ATG9A is involved in autophagy-mediated osteogenesis regulation in Ano5^–/–^ mCOBs.

To determine which initial compartments contribute to the increased autophagy, we first assessed the mRNA levels of the autophagy initiation marker UNC-51–like kinase 1 (*Ulk1*) and the phosphorylation levels at ULK1 Ser555. The results indicated no significant change in *Ulk1* mRNA levels (Supplemental Figure 5A), while the phosphorylation level at ULK1 Ser555 was upregulated in *Ano5^–/–^* mCOBs (Supplemental Figure 5B). Phosphorylation of ULK1 at Ser555 is known to initiate autophagosome formation (18). However, the application of MRT68921, an inhibitor targeting ULK1, did not significantly inhibit ALP activity of *Ano5^–/–^* mCOBs at various stages of osteogenic induction (Supplemental Figure 5, C and D).

**Figure 3:**
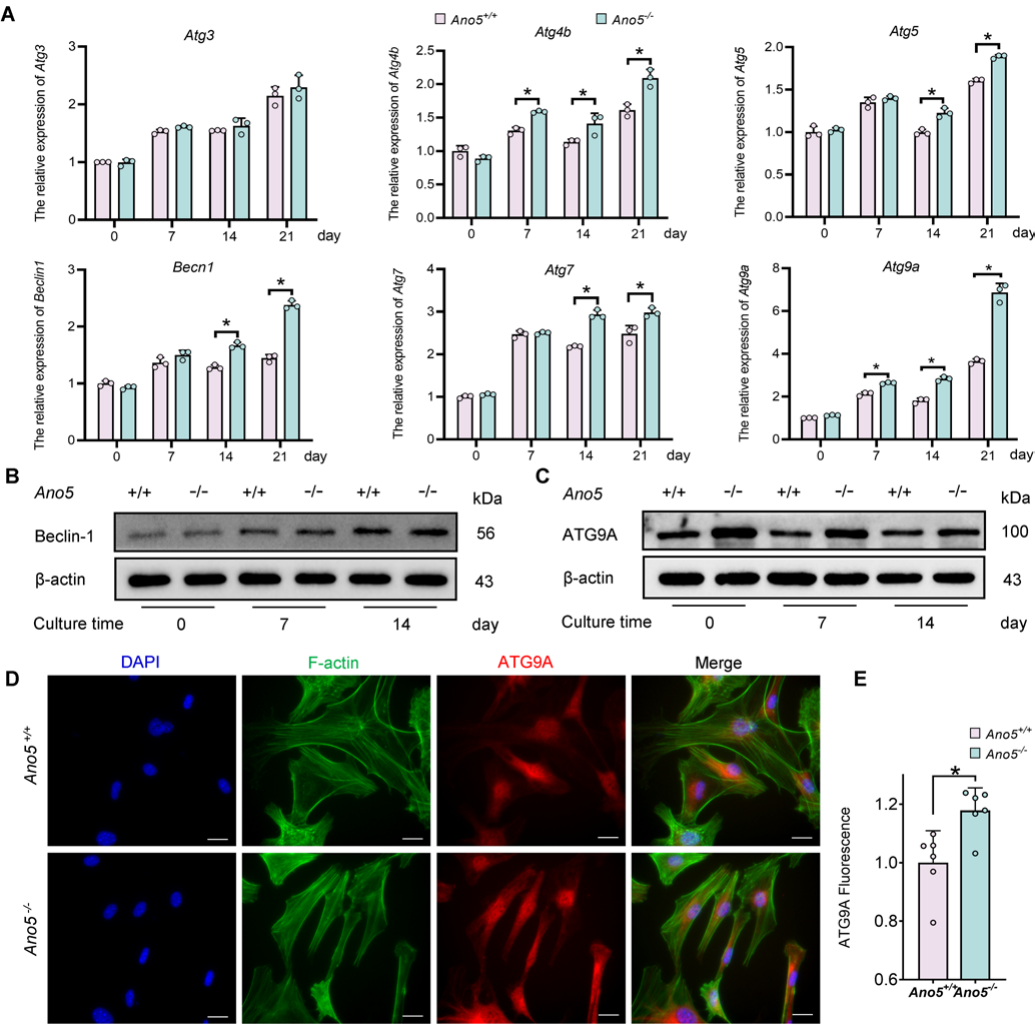
ATG9A is upregulated in *Ano5^–/–^* mCOBs. (**A**) qRT-PCR analysis of *Atg3*, *Atg4b*, *Atg5*, *Becn1*, *Atg7*, and *Atg9a* expression. (**B**) Western blot analysis of Beclin-1 protein levels. (**C**) Western blot analysis of ATG9A protein levels. (**D** and **E**) Immunofluorescence analysis showing ATG9A expression and localization in mCOBs. Scale bars: 10 μm. Data are represented as mean ± SD. *P < 0.05; NS, *P* > 0.05, as assessed by 1-way ANOVA followed by Tukey’s post hoc test.

To identify other autophagy-related proteins involved in the abnormal autophagy of *Ano5^–/–^* mCOBs, we analyzed the mRNA levels of several key genes related to autophagy, including *Atg3*, *Atg4b*, *Atg5*, *Becn1*, *Atg7*, and *Atg9a*. Among these, *Atg9a* expression exhibited the most significant upregulation in *Ano5^–/–^* mCOBs during osteogenic induction (Figure 3A). Western blot analysis further confirmed this finding, revealing that ATG9A protein expression was markedly higher in *Ano5^–/–^* mCOBs compared with *Ano5^+/+^* mCOBs, while Beclin-1 expression showed no difference between these 2 groups (Figure 3, B and C). Immunofluorescence corroborated these results, showing that ATG9A was widely distributed in the cytoplasm, with more pronounced perinuclear localization in certain cells (Figure 3, D and E). Notably, the proportion of cells exhibiting this perinuclear aggregation was markedly increased in *Ano5^–/–^* mCOBs (data not shown).

**Figure 4:**
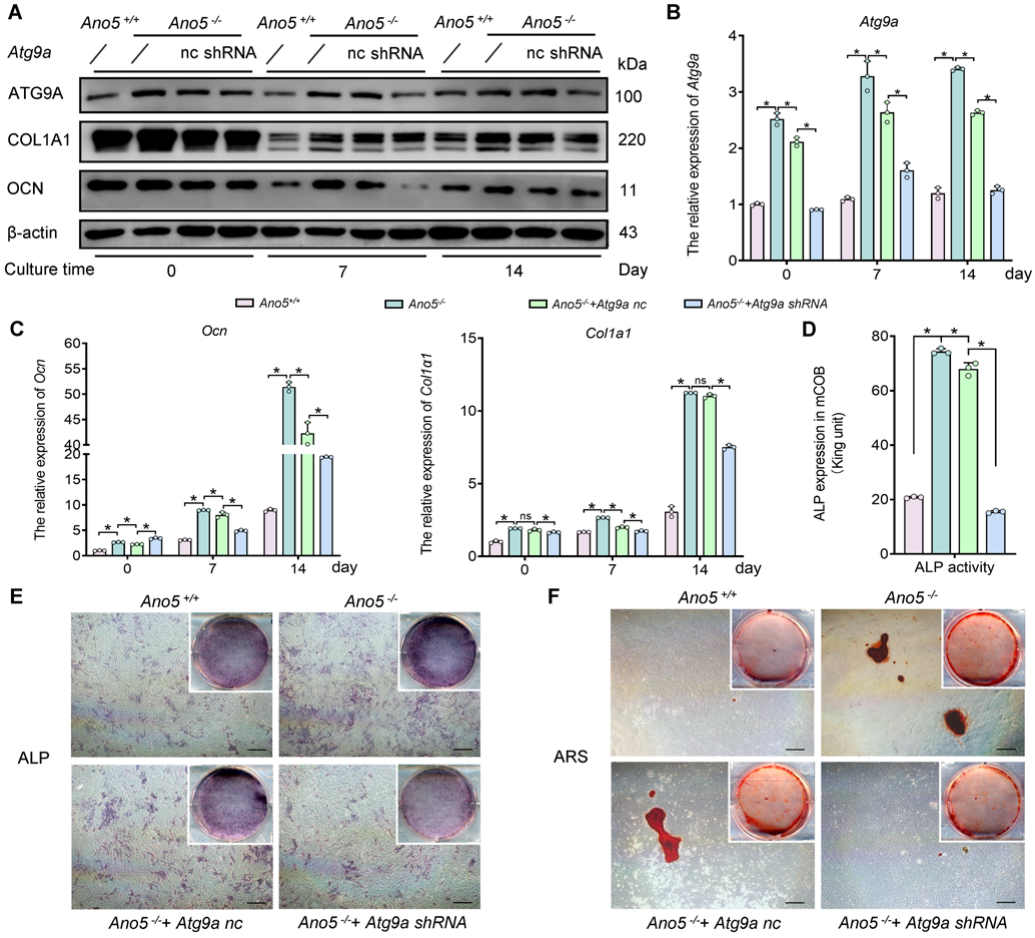
ATG9A is involved in autophagy-mediated osteogenesis regulation in *Ano5^–/–^* mCOBs. (**A–F**) For *Atg9a* knockdown, *Atg9a* shRNA was expressed in *Ano5^–/–^* mCOBs via lentiviral transfection. (**A**) Western blot analysis of ATG9A, OCN, and COL1A1 protein levels. (**B** and **C**) qRT-PCR analysis of *Atg9a*, *Ocn*, and *Col1a1* expression. (**D**) ALP activity assay. (**E**) ALP staining. Scale bars: 200 μm. (**F**) Alizarin red staining of extracellular calcium deposits. Scale bars: 200 μm. Data are represented as mean ± SD. *P < 0.05; NS, *P* > 0.05, as assessed by 1-way ANOVA followed by Tukey’s post hoc test.

Subsequently, we synthesized *Atg9a* shRNA and administered it to *Ano5^–/–^* mCOBs to investigate the effects of silencing *Atg9a*. The knockdown efficiency was confirmed by a significant reduction in both the mRNA and protein levels of ATG9A in the *Ano5^–/–^* plus *Atg9a* shRNA group during osteogenic induction (Figure 4, A and B). This suppression of autophagy was accompanied by a significant decrease in osteogenic differentiation markers, including lower mRNA and protein levels of OCN and COL1A1 compared with the control group (Figure 4, A and C). Moreover, the early stage of osteogenic differentiation was impaired, as reflected by a significant reduction in the ALP activity (Figure 4, D and E). In addition, a substantial decrease in calcium nodule formation was observed, as demonstrated by ARS (Figure 4F), indicating a disruption in the later stage of osteogenic differentiation.

Collectively, these results indicate that among the key proteins involved in autophagy initiation, ATG9A plays a particularly critical role in the abnormal autophagy associated with *Ano5* deficiency. The inhibition of ATG9A expression effectively attenuated the abnormal osteogenesis caused by *Ano5* deficiency, underscoring a strong association between ATG9A-dependent autophagy upregulation and enhanced osteogenesis in *Ano5^–/–^* mCOBs.

### Ano5 deficiency promotes osteogenesis in mCOBs via the AMPK/ATG9A pathway.

**Figure 5:**
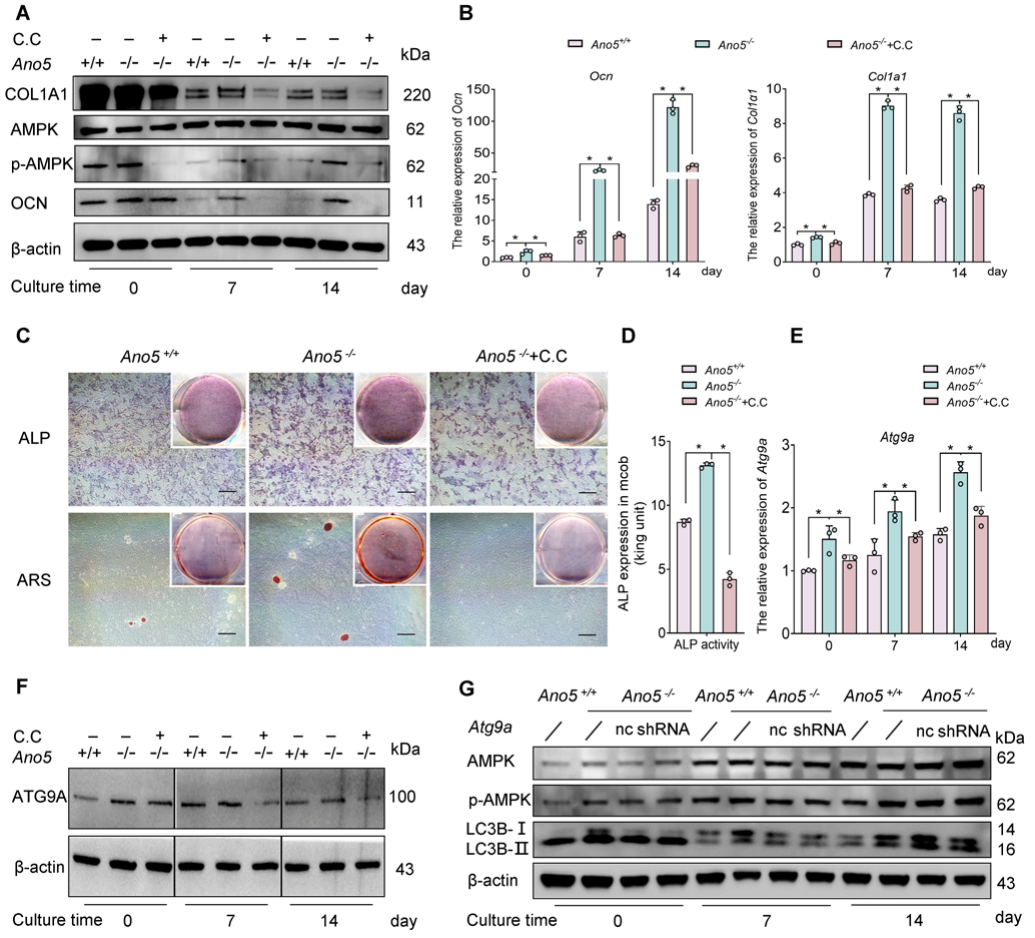
AMPK is involved in autophagy regulation in *Ano5^–/–^* mCOBs. (**A**) Schematic illustration of AMPK- and AKT-mediated regulation of autophagy. The samples were run on the same blot but the lanes are noncontiguous. (**B**) Western blot analysis of p-AMPK and AMPK protein levels. C.C treatment: (**C**–**F**) mCOBs from the *Ano5^–/–^* group were treated with C.C (an AMPK inhibitor) during osteogenic induction. (**C** and **E**) Confocal microscopy of mCOBs infected with mCherry-GFP-LC3B adenovirus, assessed on day 0 and day 14 of osteogenic induction. Representative images show autophagic activity. Scale bars: 10 μm. The zoomed-in images in **C** and **E** are magnified ×900. (**D** and **F**) Quantification of autophagosomes (mCherry^+^GFP^+^, yellow puncta) and autolysosomes (mCherry^+^GFP^–^, red puncta) in **C** and **E** respectively. Data are represented as mean ± SD. *P < 0.05; NS, *P* > 0.05, as assessed by 1-way ANOVA followed by Tukey’s post hoc test.

The PI3K/AKT/mTOR signaling pathway is widely known as a negative regulator of autophagy. However, we found that the level of p-AKT was elevated in *Ano5^–/–^* mCOBs (Supplemental Figure 6). This prompted further exploration of other autophagy regulatory pathways. Additionally, a recent study indicated that ATG9A is also regulated by the AMPK signaling pathway during autophagy progression (Figure 5A) (19). In line with these studies, Western blot analysis in our study exhibited a marked increase in phosphorylation of AMPK (p-AMPK) in *Ano5^–/–^* mCOBs (Figure 5B), which led us to hypothesize that AMPK is more likely involved in the regulation of autophagy in this model. To test this hypothesis, the AMPK inhibitor Compound C (C.C) was utilized in *Ano5^–/–^* mCOBs. As shown in Figure 5, C–F, treatment with C.C significantly downregulated the number of both autophagosomes and autolysosomes in *Ano5^–/–^* mCOBs during osteogenic induction. Notably, the reduction in autolysosomes exceeded 90%, with their total number significantly lower than that of autophagosomes, rendering autolysosomes almost undetectable within the cells treated with C.C (Figure 5, D and F).

**Figure 6:**
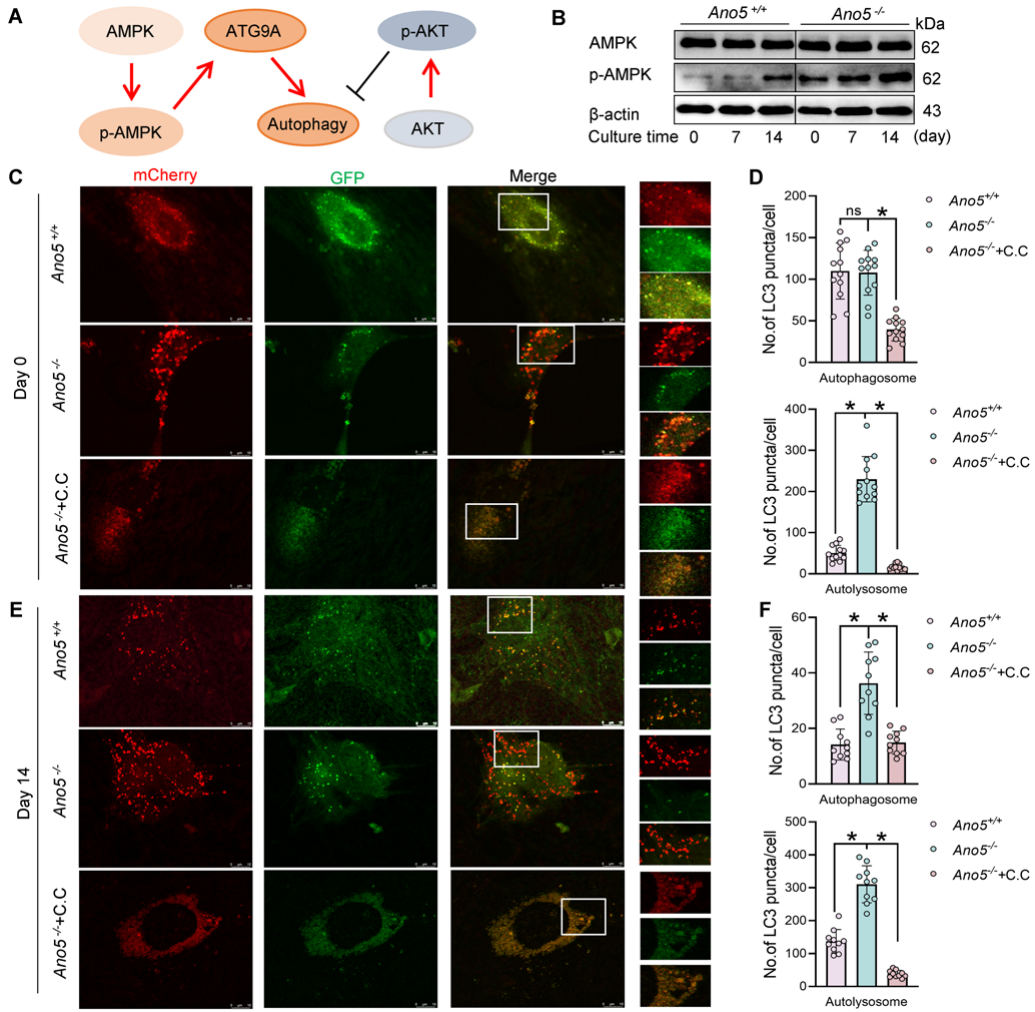
AMPK regulates osteogenic function and is positioned upstream of ATG9A. C.C treatment: (**A**–**F**) mCOBs from the *Ano5^–/–^* group were treated with C.C during osteogenic induction. (**A**) Western blot analysis of p-AMPK, AMPK, OCN, and COL1A1 protein levels. (**B**) qRT-PCR analysis of *Ocn* and *Col1a1* expression. (**C**) ALP staining and alizarin red staining. Scale bars: 200 μm. (**D**) ALP activity assay. (**E**) qRT-PCR analysis of *Atg9a* expression in mCOBs. (**F**) Western blot analysis of ATG9A protein levels. The samples were run on the same blot but the lanes are noncontiguous. (**G**) Upon *Atg9a* knockdown, Western blot analysis of LC3B-II, p-AMPK, and AMPK protein levels. Data are represented as mean ± SD. *P < 0.05; NS, *P* > 0.05, as assessed by 1-way ANOVA followed by Tukey’s post hoc test.

In terms of osteogenic differentiation, the inhibition of AMPK by C.C led to a significant rescue of the enhanced osteogenesis observed in *Ano5^–/–^* mCOBs. Protein and mRNA levels of key osteogenic markers, including OCN and COL1A1, were markedly reduced compared with the untreated *Ano5^–/–^* group (Figure 6, A and B). Additionally, the ALP activity was significantly downregulated in the *Ano5^–/–^* plus C.C group, suggesting a restoration of osteogenic potential (Figure 6, C and D). This was further corroborated by decreased calcium nodule formation, as shown by ARS (Figure 6C).

**Figure 7:**
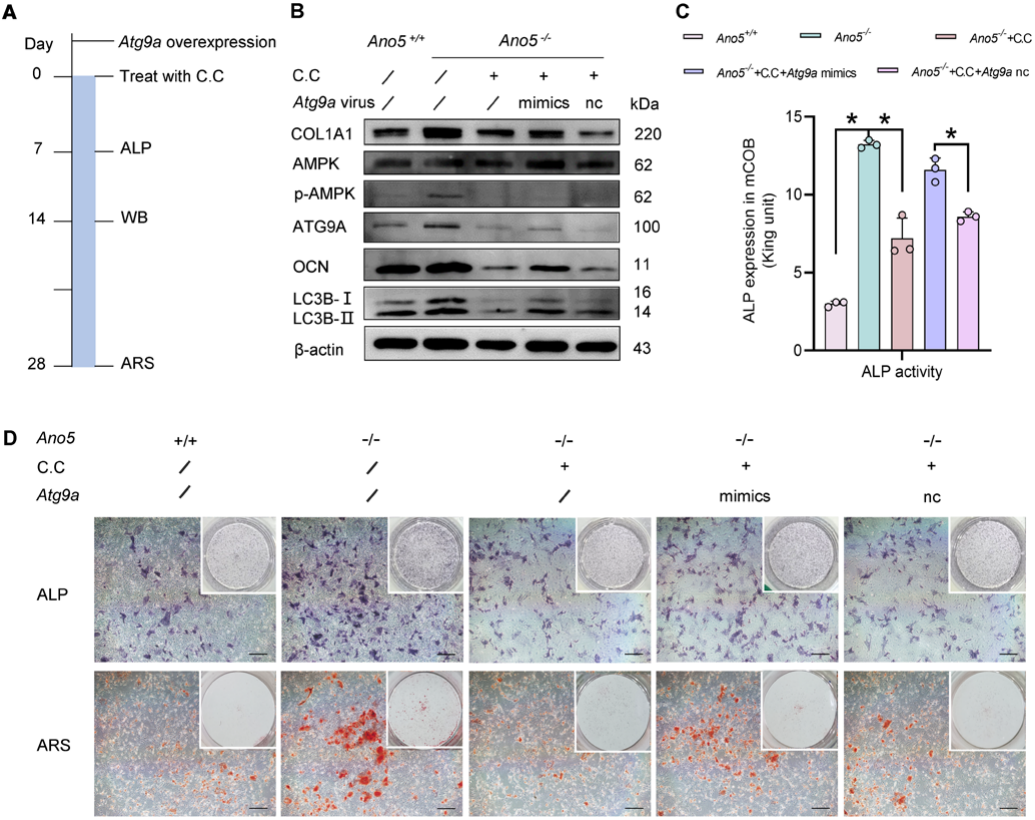
*Ano5* deficiency promotes osteogenesis in mCOBs via the AMPK/ATG9A pathway. Following *Atg9a* overexpression combined with C.C treatment: (**A**) mCOBs were first selected for *Atg9a* overexpression, followed by C.C treatment. (**B**) Western blot analysis of p-AMPK, AMPK, ATG9A, LC3B-II, OCN, and COL1A1 protein levels. (**C**) ALP activity assay. (**D**) ALP staining and alizarin red staining. Scale bars: 200 μm. Data are represented as mean ± SD. *P < 0.05; NS, *P* > 0.05, as assessed by 1-way ANOVA followed by Tukey’s post hoc test.

Recent research has shown that ATG9A can be regulated by AMPK directly, independently of any other initial complexes (19). To verify whether the regulation of autophagy by AMPK is mediated through ATG9A, we measured ATG9A levels in the *Ano5^–/–^* plus C.C group. Western blot analysis showed that AMPK inhibition by C.C resulted in significantly decreased ATG9A expression (Figure 6, E and F). We further assessed the relationship between ATG9A and AMPK by using *Atg9a* shRNA to knock down ATG9A expression in *Ano5^–/–^* mCOBs. The results demonstrated that downregulation of ATG9A could reduce autophagy levels, as evidenced by the reduction in LC3B-II expression, but did not change the expression of p-AMPK (Figure 6G). Furthermore, we applied *Atg9a* mimics to the *Ano5^–/–^* plus C.C group and assessed their osteogenic differentiation. The workflow of the rescue experiment is presented in Figure 7A. The results showed that the overexpression of ATG9A substantially rescued the impaired autophagy and osteogenic capacity caused by C.C during osteogenic induction in *Ano5^–/–^* mCOBs, as evidenced by partially restored protein levels of LC3B-II, OCN, and COL1A1, while p-AMPK expression was still at a low level (Figure 7B). Additionally, the ALP activity increased, and calcium nodule formation improved, as shown by ALP/ARS staining and ALP activity quantification (Figure 7, C and D).

Taken together, these findings provide strong evidence that ANO5 modulates autophagy, at least in part, via the AMPK/ATG9A signaling pathway, and this modulation plays a crucial role in the regulation of osteogenesis in mCOBs.

### The application of 3-MA in vivo alleviated the abnormal bone formation.

**Figure 8:**
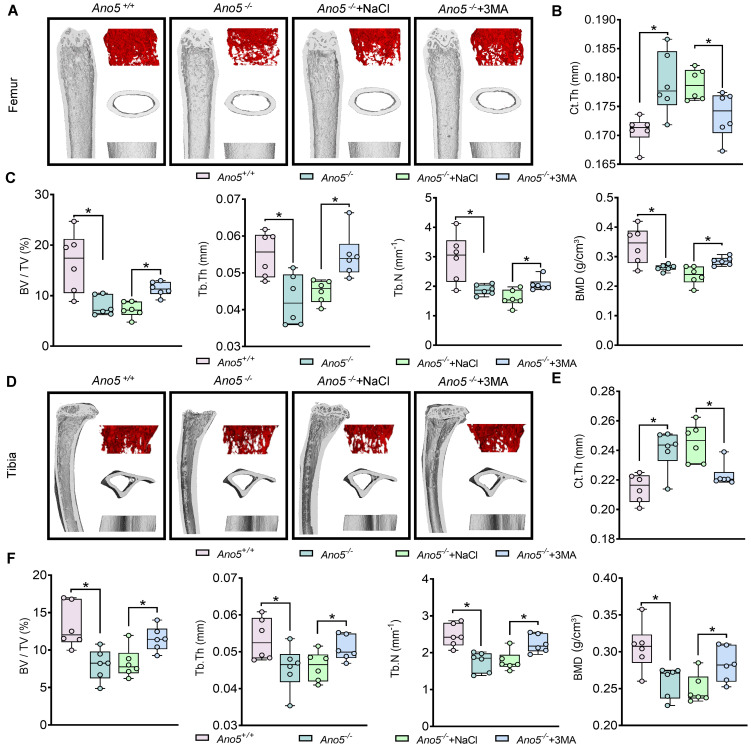
In vivo administration of 3-MA alleviated the abnormal phenotype in *Ano5^–/–^* mice. Femoral trabecular bone mass was assessed by micro-CT. Representative 3D reconstruction (**A**) and relative quantification (**B** and **C**) are displayed. Tibial trabecular bone mass was assessed by micro-CT. Representative 3D reconstruction (**D**) and relative quantification (**E** and **F**) are displayed (*n* = 6 per group). Ct.Th, cortical bone thickness; BV/TV, trabecular bone volume/total volume; Tb.Th, trabecular thickness; Tb.N, trabecular number per cubic millimeter; BMD, trabecular bone mineral density. Data are represented as mean ± SD. *P < 0.05; NS, *P* > 0.05, as assessed by 1-way ANOVA followed by Tukey’s post hoc test.

3-MA, a well-established autophagy inhibitor, is commonly used in vivo to block autophagy progression in animal models (20). Therefore, we selected 3-MA as the intervention drug for in vivo administration. The results showed that *Ano5^–/–^* mice exhibited increased cortical bone thickness (Ct.Th) in the femurs and tibiae compared with the *Ano5*^+/+^ group (Figure 8 and Supplemental Figure 7). Concurrently, the trabecular bone volume fraction (BV/TV), bone mineral density (BMD), trabecular thickness (Tb.Th), and trabecular number (Tb.N) were all significantly reduced in the *Ano5^–/–^* group relative to the *Ano5*^+/+^ controls. Following 3-MA treatment, Ct.Th in the femurs and tibiae of *Ano5^–/–^* mice decreased compared with the *Ano5^–/–^* plus NaCl group. Simultaneously, BV/TV, BMD, Tb.Th, and Tb.N all demonstrated significant increases after treatment (Figure 8, A–F).

**Figure 9:**
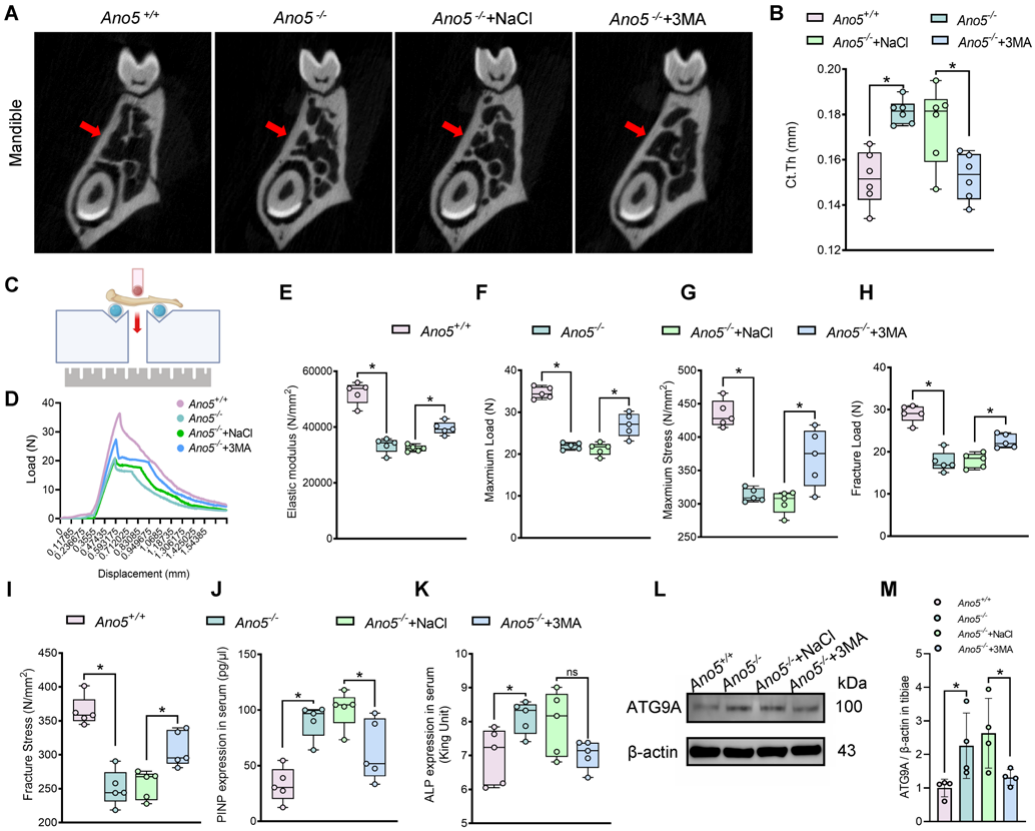
In vivo administration of 3-MA alleviated the abnormal phenotype in *Ano5^–/–^* mice. (**A** and **B**) Mandibles were assessed by micro-CT, with the red arrows representing the cortical bone on the palatal side of the first molar region (*n* = 6 per group). (**C**) Schematic representation of the 3-point bending test used to evaluate the biomechanical function of tibiae. (**D**–**I**) The biomechanical properties of tibiae were assessed through the 3-point bending test (*n* = 5 per group). (**J** and **K**) Serum levels of PINP and ALP (*n* = 5 per group). (**L** and **M**) The expression levels of ATG9A. Data are represented as mean ± SD. *P < 0.05; NS, *P* > 0.05, as assessed by 1-way ANOVA followed by Tukey’s post hoc test.

Additionally, Ct.Th on the palatal side of the first molar region of the mandible was significantly greater in the *Ano5^–/–^* group; however, this thickness markedly decreased after 3-MA treatment (Figure 9, A and B). The 3-point bending experiment revealed a significant reduction in the elastic modulus of the tibiae in the *Ano5^–/–^* group. Both the load-bearing capacity and stress tolerance of the tibiae in the *Ano5^–/–^* group were markedly lower compared with the *Ano5*^+/+^ group. Following treatment with 3-MA, the tibiae demonstrated a substantial improvement in vertical load tolerance and a higher elastic modulus (Figure 9, C–I). Serological analysis showed that serum levels of ALP and procollagen type I N-terminal propeptide (PINP) were significantly higher in *Ano5^–/–^* mice than in *Ano5*^+/+^ mice. Upon 3-MA administration, ALP levels in *Ano5^–/–^* serum showed a decreasing trend without statistical significance, whereas PINP levels were significantly reduced (Figure 9, J and K). We subsequently measured the levels of ATG9A in the tibiae, and the results showed that the ATG9A levels in the *Ano5^–/–^* group were higher than those in the *Ano5*^+/+^ group. After applying 3-MA to inhibit autophagy, the ATG9A levels significantly decreased (Figure 9, L and M, and Supplemental Figure 8, A and B). These results further confirm that ATG9A, previously validated in vitro, also exerts an important role in vivo in regulating bone formation.

## Discussion

In this study, we found that *Ano5* deficiency enhances autophagic flux via the AMPK/ATG9A/autophagy axis, which, in turn, promotes osteogenic differentiation in mCOBs. Our findings provide partial insights into the underlying mechanisms of the clinical manifestations observed in GDD, suggesting that interventions aimed at modulating autophagy levels may play a foundational role in the treatment or prevention of GDD-associated damage.

*ANO5* was first identified as the causative gene for GDD by Tsutmumi et al. in 2003 (21). With the increasing number of GDD cases globally, more dominant mutations in different loci of *ANO5* have been uncovered, all leading to GDD-like pathological manifestations (5, 22). Furthermore, among all reported GDD individuals from 25 families, 84% have been identified with dominant *ANO5* mutations through genetic testing. Another 4 families have not undergone genetic screening, meaning the actual proportion may be higher. As most of these mutations are located in exons 11 and 12 of *ANO5*, and given the high conservation of ANO5 between humans and mice, our group successfully established an *Ano5*-knockout mouse model targeting exons 11 and 12, exhibiting a similar skeletal phenotype to that of GDD, with increased serum ALP levels in vivo and enhanced osteogenic capacity of mCOBs in vitro. Subsequently, we sought to investigate the underlying mechanism of this change. Many key processes in osteoblasts, such as autophagy, proliferation, and differentiation, are closely linked to cellular osteogenic activity. Through clustering analysis of proteins interacting with ANO5, it was revealed that these proteins are primarily involved in the following functional domains: protein translation and processing, energy metabolism, mitochondrial function, membrane transport and vesicle formation, protein degradation, signal transduction, and nucleic acid metabolism (11). Some of these functions are closely related to autophagy. Additionally, Foltz et al. found that *Ano5* knockout leads to the accumulation of annexin A2, which has been shown to enhance autophagy in certain diseases (12, 13). However, this study also demonstrated a decrease in the expression of annexin A1, annexin A5, and annexin A6, which contrasts with the findings of Su et al., suggesting that the expression patterns of these annexin proteins may not consistently align (12, 17). This discrepancy could be related to the differences in the knockout sites of *Ano5* in different models. Moreover, the relationship between the TMEM16 protein family and autophagy has been demonstrated by other members as well (23, 24). Recent research found that ANO1, another member of the TMEM16 family, negatively regulates autophagy by inhibiting the formation of the Bcl-2–p62 complex (23). Prompted by these studies and the widely accepted inherent connection between autophagy and osteogenesis, we conducted experiments in cultured mCOBs in vitro and observed significant upregulation in the levels of the classical autophagy marker LC3B-II (also known as ATG8 in yeast), while the other marker, p62, remained unchanged.

Autophagy is a dynamic process involving the formation and degradation of autophagic vesicles. In autophagy assays, LC3B-II and p62 serve as markers for these 2 steps, respectively. Typically, an increase in LC3B-II accompanied by a decrease in p62 is considered indicative of enhanced autophagic flux (25). Therefore, changes in LC3B-II expression alone cannot determine whether the accumulation of it is due to enhanced autophagic vesicle formation or impaired degradation. To differentiate between these, CQ was applied to block autophagic degradation, enabling the observation of total LC3B-II production over time. The results showed a more pronounced accumulation of LC3B-II in *Ano5^–/–^* mCOBs, suggesting that autophagic vesicle formation was more active compared with *Ano5*^+/+^ mCOBs. Furthermore, the results from TEM revealed a greater increase in the number of autolysosomes in *Ano5^–/–^* mCOBs rather than autophagosomes, which was further corroborated by mCherry-GFP-LC3B experiments. We hypothesized that autophagosomes rapidly fused with lysosomes upon formation to generate autolysosomes, prompting the evaluation of genes involved in the fusion process, and the results aligned with the hypothesis. Previous studies have demonstrated that the deficiency of CDKN1B, a cyclin-dependent kinase inhibitor, leads to lysosomal dysfunction with an acidity defect, impairing degradative activity, ultimately causing abnormal autolysosome accumulation (26). Therefore, Lyso-Tracker was utilized to trace lysosomes in mCOBs, as this probe specifically marks acidic structures. Our results demonstrated a substantially higher number of acidic lysosomes in the *Ano5^–/–^* group compared with the *Ano5*^+/+^ group. In conclusion, our study demonstrates that autophagic flux is significantly upregulated in *Ano5^–/–^* mCOBs, characterized by a marked increase in autolysosomes. Finally, the increased expression of LC3B-II detected in femurs, tibiae, and mandibles from mice underscores the clinical relevance of our findings obtained in vitro.

Autophagy and osteogenic differentiation are closely interconnected. Appropriate levels of autophagy are crucial for cellular osteogenic differentiation and the formation of bone matrix. Abnormal changes in autophagy levels have been observed in various skeletal diseases, including intervertebral disc degeneration, osteoarthritis, osteoporosis, and osteosclerosis, closely linked to pathological states of the skeleton (27, 28). Studies have shown that the application of autophagy modulators exerts a regulatory effect beneficial for bone protection (29). In our study, although the differences in autophagy of the *Ano5^–/–^* group have been confirmed both in vitro and in vivo, it remains unclear whether these autophagic differences are related to the abnormal osteogenic phenotype. Current research predominantly suggests that autophagy plays a role in regulating osteoblast differentiation (30–32). However, some studies have reported feedback regulation from osteogenesis to autophagy. For instance, matrix vesicles containing annexin A5 could mediate mineralization in precursor osteoblasts and subsequently activate autophagy (17); however, it seems that it is the role of annexin A5, rather than changes in cellular mineralization, that is likely responsible for the changes in autophagy. In light of these insights, we initially hypothesized that autophagy might play a regulatory role in osteogenic differentiation of *Ano5^–/–^* mCOBs. To test this, 3-MA, a classical early-stage autophagy inhibitor, was utilized to observe the effects of autophagy inhibition on osteogenic activity. Results indicated that inhibiting autophagic vesicle formation significantly reduced osteogenic activity of osteoblasts. Moreover, Yan et al. found that LC3-positive autophagosomes carrying the minerals can be directly secreted into the extracellular space, thereby promoting calcification (14). To verify whether the enhanced osteogenic capacity observed in *Ano5^–/–^* mCOBs was due to the secretion of autophagosomes, CQ was applied to inhibit autophagic degradation, which allowed autophagosome accumulation to continue while blocking their degradation. The results showed a significant decrease in osteogenic capacity after CQ treatment, indicating that the enhanced osteogenesis in *Ano5^–/–^* mCOBs is likely due to excessive autophagic flux rather than the accumulation or secretion of autophagosomes.

Due to the intrinsic link between autophagy and osteogenesis, further investigation into upstream regulatory mechanisms is warranted. ULK1, also known as ATG1, is a key kinase in the autophagy initiation complex, with phosphorylation at Ser555 playing a critical role in autophagosome biogenesis (33). However, despite the observed elevated expression of p-ULK1 Ser555, no significant changes in ALP activity were detected during osteogenesis in *Ano5^–/–^* mCOBs treated with the ULK1 inhibitor MRT68921. This suggests that in the abnormal autophagy caused by *Ano5* deficiency, other proteins may play early regulatory roles. Among these, *Atg5*, *Becn1*, and *Atg7* are frequently reported (34, 35). We therefore analyzed the gene and protein levels of several key autophagy-related genes. Interestingly, the expression of Beclin-1, a critical protein required for canonical autophagy pathway, remained unchanged in *Ano5^–/–^* mCOBs. This observation is consistent with the findings of other researchers suggesting that autophagy can be initiated through non-canonical pathways, thus bypassing the Beclin-1–ATG14-VPS34 complex (36). Furthermore, during our examination, ATG9A exhibited a notable increase in expression. As the only multitransmembrane protein among autophagy-related proteins, ATG9A is involved in phospholipid transport and assembly via its phospholipid scramblase function (37, 38). The research on the proximity interaction network of ATG9A revealed that under non-starvation conditions, ATG9A can act independently of ULK1 (39), which is consistent with our previous findings that ULK1 might play an unnecessary role in our model. Active ATG9A typically localizes around the nucleus, attached to vesicle puncta near the endoplasmic reticulum and Golgi membranes. However, only 10%–20% of these puncta contribute to phagophore formation and subsequent autophagic vesicle biogenesis. David et al. demonstrated through dual-color imaging that ATG9A puncta that fail to recruit LC3B-II have shorter lifespans, while those that do persist longer, aiding the formation of larger phagophores and autophagic vesicles (40). The increase in ATG9A-positive puncta is likely responsible for the elevated autophagy levels, as it enhances the probability of interaction between ATG9A puncta and LC3B-II. Upon knocking down *Atg9a* in *Ano5^–/–^* mCOBs, both autophagy levels and osteogenic activity were reduced.

Previous studies have reported that ULK1 phosphorylation regulates ATG9A localization in an AMPK-dependent manner, suggesting AMPK’s involvement in ATG9A positioning (41). AMPK is recognized as a key regulator of autophagy, predominantly known for promoting autophagy. However, recent research showed that glucose starvation, via AMPK activation, reduced mature autolysosome numbers by causing an accumulation of LC3-positive autophagosomes, rather than affecting autophagosome formation (42). Additionally, increased levels of both p-AMPK and ATG9A were observed in a study on acute kidney tubular necrosis (43). In another study involving an IRP2-knockout model, impaired AMPK activation was linked to defective ATG9A endosomal trafficking (44), although the interaction between AMPK and ATG9A was not explored further. These findings imply a potential regulatory interaction between AMPK and ATG9A. Moreover, previous reports indicate that under hypoxic stress, activated AMPK can bypass ULK1 to directly regulate ATG9A and promote autophagic vesicle formation (45). In our study, ULK1 inhibition did not lead to significant changes in osteogenesis of *Ano5^–/–^* mCOBs, and after observing upregulation of AMPK signaling, we treated *Ano5^–/–^* mCOBs with the AMPK inhibitor C.C. This treatment resulted in autolysosome reduction, reduced ATG9A expression, and decreased osteogenic capacity. However, this alone does not confirm whether AMPK and ATG9A function in a linear pathway or independently. To investigate this, we knocked down *Atg9a* and observed no effect on AMPK activation. However, inhibition of AMPK led to downregulation of ATG9A expression. When mCOBs were cotreated with C.C and *Atg9a* overexpression mimics, the osteogenic impairment induced by C.C was alleviated. These results suggest that AMPK exerts a positive regulatory effect on ATG9A, which may, in turn, influence the bone phenotype in GDD.

To evaluate whether autophagy modulation could reverse the abnormal osteogenic phenotype in *Ano5^–/–^* mice, we administered the autophagy inhibitor 3-MA. As previously reported and observed in patients, abnormal cortical bone thickening may lead to degenerative degradation of trabecular bone and increased bone fragility (46). The results showed a significant improvement in bone phenotype metrics compared with the *Ano5^–/–^* mice and provide substantial encouragement and support for our exploration.

Our study highlights a key distinction in bone disease research; while mechanisms of osteopetrosis are primarily modeled around osteoclast dysfunction, we demonstrate that osteoblast functional gain can also lead to abnormal bone hardening. Dominant mutations in ANO5, particularly deleterious ones, lead to significant skeletal changes. Previous studies have shown that ANO5 protein instability, exacerbated by deleterious mutations, results in proteasomal degradation (1). In our mouse model, we observed similar changes in skeletal structure. Furthermore, ANO5 deficiency enhances autophagy in osteoblasts, which results in excessive osteogenesis. The marked increase in autophagic vesicles suggests that more effective degradation products are involved in bone formation. Although the exact mechanisms remain undefined, our in vivo results clearly indicate that autophagy plays a critical role in GDD pathogenesis. While autophagy activation has been explored in treating diseases like osteoporosis and osteoarthritis (29), our findings suggest that autophagy inhibition could also be a therapeutic strategy for bone diseases with sclerotic features. Given the importance of autophagy, targeting specific autophagy-related proteins, such as ATG9A, could offer a more effective approach for modulating autophagy in bone diseases.

In conclusion, our study demonstrates the intricate involvement of the AMPK/ATG9A/autophagy axis in regulating osteogenesis under *Ano5* deficiency. The use of autophagy inhibitors, such as 3-MA, highlights a potential therapeutic strategy for addressing autophagic dysregulation in GDD-related skeletal pathologies. Further exploration of the molecular interactions between autophagy and osteogenesis could lead to more precise therapeutic interventions for GDD and related disorders.

## Methods

### Sex as a biological variable.

Sex was not considered as a biological variable. In the previous study, we examined male and female mice, and similar findings were observed in both sexes.

### Animal model.

The generation of *Ano5^–/–^* mice has been described previously (Supplemental Figure 1A) (10). Mice were housed in a specific pathogen–free environment with a 12-hour light/dark cycle, 45% to 65% relative humidity, and an ambient temperature of 20°C to 24°C. Mice had ad libitum access to tap water and standard rodent food.

### Cell isolation and culture.

The isolation and culture of mCOBs have been described in our previous research (10), and a 0.2 cm tail sample was collected for genotyping (Supplemental Figure 1B). Cells at passage 2 (P2) were seeded at a density of 2 × 10^5^ cells/well in 6-well plates and cultured overnight. Once the cell density reached 90%, the culture was designated as day 0, and osteogenic induction was initiated. The osteogenic induction medium consisted of α-MEM (Gibco/Invitrogen) supplemented with 10% fetal bovine serum (FBS; Gibco/Invitrogen), 1% penicillin-streptomycin (Gibco/Invitrogen), 100 μM ascorbic acid (Sigma-Aldrich), 10 mM β-glycerophosphate (Sigma-Aldrich), and 10 nM dexamethasone. The medium was refreshed every 2 days during the induction process.

### Lentiviral transfection.

mCOBs from P2 were selected for transfection. Transfection was performed when the cell confluence reached 30%, using an MOI value optimized in preliminary experiments to ensure a transfection efficiency exceeding 90%. After 2 days of transfection, cells were subjected to selection with 2 μM puromycin for 2 days. Subsequent experiments were conducted when the cell confluence exceeded 90%. The following lentiviruses were used in this study: *Atg9a* shRNA (Genechem) and *Atg9a* mimics (Genechem).

### Western blotting.

Protein extraction and sodium dodecyl sulfate-polyacrylamide gel electrophoresis were performed as previously described (17). The resolved proteins on the gel were transferred to a polyvinylidene difluoride membrane. The membrane was blocked with 5% nonfat milk for 1 hour and then incubated overnight with the primary antibody. The following day, a horseradish peroxidase–conjugated secondary antibody, diluted 1:5000, was incubated with the membrane at room temperature for 1 hour. Finally, images were acquired using the ChemiDoc Touch imaging system (Bio-Rad). β-Actin was utilized as the internal control for detection. Antibodies used in the study are listed in Supplemental Table 1.

### TEM.

After 14 days of osteogenic induction, the medium was discarded and 2.5% glutaraldehyde solution at ambient temperature was added to fix the samples for 5 minutes, protected from light. A transmission electron microscope (JEOL) was utilized to capture images of samples. The number of autophagosome and autolysosome was calculated using ImageJ software (NIH).

### Autophagic flux assay with mCherry-GFP-LC3B adenoviral transfection.

mCOBs were seeded at a density of 1 × 10^4^ cells per dish in laser confocal 15 mm cell culture dishes. After a 6-hour incubation to allow cell adhesion, the medium was replaced. The volume of mCherry-GFP-LC3B viral solution (Beyotime) required for infection was calculated based on the cell count, and subsequently diluted in 1 mL of culture medium before being added to the dishes. After 72 hours of infection, the dishes were transferred to a laser confocal microscope (Leica Microsystems) for observation. Fluorescence microscope images were analyzed using Image Pro Plus image processing software compatible with 32-bit Windows (ipwin 32) (Media Cybernetics). In the merged images, yellow fluorescent puncta indicate autophagosomes, while red fluorescent puncta represent autolysosomes.

### Lysosome analysis.

Lyso-Tracker Red stock solution (Beyotime) was diluted in α-MEM at a ratio of 1:13,333 to prepare a working solution with a final concentration of 75 nM, prewarmed in a 37°C incubator. After removing the culture medium, the prewarmed Lyso-Tracker Red working solution was added, and cells were incubated at 37°C for 60 minutes. After incubation, the staining solution was removed and replaced with fresh culture medium. Cells were observed and photographed using a fluorescence microscope (Zeiss) (maximum excitation wavelength: 577 nm; maximum emission wavelength: 590 nm). The images were analyzed using the ipwin 32 image processing software.

### Quantitative real-time PCR.

The procedures were conducted as we described in a previous study (17). Cells were lysed on ice using TRIzol (Invitrogen) for total RNA extraction. cDNA was synthesized from 1 μg of RNA using the Super RT cDNA Synthesis Kit (CWbio). Quantitative real-time PCR (qRT-PCR) was performed using a low-ROX SYBR Premix (CWbio) on a Bio-Rad real-time PCR machine, with β-actin (*Actb*) as an internal control. The relative expression levels of genes were calculated using the 2^–ΔΔCt^ method. The primer sequences and names used in this study are listed in Supplemental Table 2.

### ALP staining.

Following osteogenic induction in mCOBs, ALP staining was performed using an ALP staining kit (Beyotime), following the product protocol. Briefly, cells were fixed with 4% paraformaldehyde for 20 minutes, washed, and incubated with the prepared working solution at room temperature for 1 hour. The staining reaction was terminated by double-distilled water, and images were acquired under ×40 magnification, along with macroscopic observation of different regions.

### Immunofluorescence of cells.

Cells were washed 3 times with PBS for 5 minutes each and then fixed with 4% paraformaldehyde for 20 minutes at room temperature. Then, cells were blocked using a blocking solution (Beyotime) for 10 minutes. Primary antibody (anti-ATG9A; BOSTER) was diluted 1:200 in antibody dilution buffer and incubated overnight at 4°C. After overnight incubation, cells were incubated with Cy3-conjugated secondary antibodies (Abclonal) (diluted 1:200) for 1 hour at room temperature, protected from light. F-actin filaments were labeled with FITC-conjugated phalloidin (Sigma-Aldrich), protected from light. Nuclei were labeled with 4′,6-diamidino-2-phenylindole (DAPI) (Sigma-Aldrich), protected from light. Cells were observed under a fluorescence microscope (Zeiss).

### ALP activity assay.

Following osteogenic induction differentiation, mCOBs were assessed using the ALP Activity Assay kit (Nanjing Jiancheng Bioengineering Institute), following the manufacturer’s protocol. Briefly, proteins were collected using simple RIPA lysis buffer according to a protein detection method described previously (17). The collected proteins underwent a chromogenic reaction with the phosphate substrate from the kit, followed by incubation at 37°C for 30 minutes. ALP activity was determined via colorimetric analysis. The calculation formula is as follows: ALP activity (King units/g protein) = (OD of sample – OD of blank)/(OD of standard – OD of blank) × (0.01 mg/mL/protein concentration mg/grams of protein/mL).

### ARS.

Following osteogenic induction differentiation, mCOBs were subjected to ARS (Beyotime) according to the manufacturer’s protocol. The cells were fixed with a 4% formaldehyde solution at 4°C for 30 minutes. After discarding the fixation solution, 1 mL of alizarin red working solution was added to each well and incubated in the dark for 10 minutes. Following the removal of the staining solution, images were captured at a magnification of ×40, along with gross views, to assess the number and area of mineralized nodules in each region.

### Injection of 3-MA in vivo.

Twelve-week-old male *Ano5*^+/+^ and *Ano5^–/–^* littermates were divided into 4 groups: *Ano5*^+/+^, *Ano5^–/–^*, *Ano5^–/–^* plus NaCl (saline), and *Ano5^–/–^* plus 3-MA. The 3-MA powder (MedChemExpress) was diluted in saline to a final concentration of 10 μM. Intraperitoneal injections were administered every other day for 4 weeks with a dosage at 1.5 mg per 100 g of body weight. Tissue samples were collected at the 16th week.

### Micro-computed tomography.

The femur, tibia, and mandible from mice were collected and analyzed using a micro-computed tomography (micro-CT) scanner (Inve on CT, Siemens). The tissues were subjected to microfocus x-ray projections from various angles and the scans were performed to reconstruct volumetric images using algorithms, enabling dynamic analysis of bone tissue morphology. The CT scan voxel size was set to 15 μm.

For mandible trabecular bone analysis, the first molar region was selected for comparison. For the femur and tibia, parameters such as trabecular and cortical bone were measured over 100 consecutive slices of images taken from the region near the growth plate of the knee. BMD was calculated using Hounsfield units (HU) and calibrated CT data. Image processing, including 3-dimensional reconstruction and quantitative analysis, was performed using an interactive medical imaging control system (Mimics Medical 21.0, Materialise).

### Serum levels of bone generation markers.

The levels of bone generation markers in the serum of *Ano5*^–/–^ mice after injection of 3-MA were measured using the respective enzyme-linked immunosorbent assays (ELISA) according to the manufacturer’s instructions. Levels of ALP were measured by the ALP Activity Assay kit (Nanjing Jiancheng Bioengineering Institute). The concentrations of PINP were determined using the Mouse PINP ELISA Kit (Elabscience).

### Three-point bending experiment.

Using a universal testing machine (AG-X Plus, Shimadzu), the tibia was placed on a support with a span of 12.5 mm, while a load-sensor-equipped head was positioned at the center of the tibia. A 3-point bending test was conducted at a displacement rate of 1 mm/min until the tibia fractured. The corresponding load-displacement curve, elastic modulus, fracture stress, and maximum stress were recorded. The diameter of each tibial fracture end was measured with a vernier caliper, and the average of 3 measurements was taken. The load was calculated according to the following formula: *S* = 8*F* × *L*/(*D*³ × 3.1416), where *S* is stress, *F* is load, *D* is diameter, and *L* is span.

### Histological analysis.

The methods and procedures for hematoxylin and eosin (H&E) staining and immunohistochemical (IHC) staining were performed as previously described (46). Briefly, bone tissues were fixed in 4% paraformaldehyde, decalcified in EDTA solution, embedded in paraffin, and sectioned at 5 μm thickness. For H&E staining, sections were stained with H&E to visualize general histological structures. For IHC staining, sections were incubated with primary antibodies targeting specific proteins, followed by detection using an appropriate secondary antibody and chromogenic substrate. The stained sections were examined and imaged under a light microscope.

### Statistics.

All data are expressed as the mean ± standard deviation (SD). Data analysis was performed using R statistical software (version 4.2.0, R Foundation) and Prism (version 10, GraphPad Software). Comparisons between 2 groups were conducted using a 2-tailed Student’s *t* test. For comparisons involving more than 2 groups, 1-way analysis of variance (ANOVA) was employed, followed by Tukey’s post hoc test to determine significant differences between groups. All statistical tests were 2-sided with a significance level set at 0.05. Prior to analysis, normality and homogeneity of variance were tested to ensure the appropriateness of the statistical methods used.

### Study approval.

Animal breeding and all experimental procedures, including defined humane endpoints, were approved by the Institutional Animal Care and Use Committee of Beijing Stomatological Hospital (approval number: KQYY-201611-001).

### Data availability.

The data that support the findings of this study are available from the corresponding author upon reasonable request. Values for all data points in graphs are reported in the Supporting Data Values file.

## Author contributions

YH, HL, and SZ initiated the project and designed the experiments. SZ conducted the majority of the experiments, including their analysis and interpretation, with assistance from SW, SL, XL, MZ, HX, and XW. Mouse experiments were conducted by SZ, SW, SL, and MZ. Technical and material support was provided by YH and HL. SZ wrote the manuscript, which was reviewed by all authors.

## Supplementary Material

Supplemental data

Unedited blot and gel images

Supporting data values

## Version 1 03/11/2025

In-Press Preview

## Version 2 04/22/2025

Electronic publication
