# Supplementary material for: Anoctamin-5 deficiency enhances ATG9A-dependent autophagy, inducing osteogenesis and gnathodiaphyseal dysplasia–like bone formation
Source: JCI Insight. 2025 Mar 11;10(8):e189817. doi: 10.1172/jci.insight.189817 (PMC12016930; doi:10.1172/jci.insight.189817)
Supplement: Supplemental data [file jciinsight-10-189817-s158.pdf]

Supplementary Data

Anoctamin 5 Deficiency Enhances ATG9A-Dependent Autophagy, Inducing Osteogenesis and GDD-like Bone Formation

Shuai Zhang[1], Shengnan Wang[1], Sirui Liu[1], Xiu Liu[1], Mingyue Zhang[1], Huichong Xu[1], Xiaoyu Wang[1], Hongyu Li[1*], Ying Hu[1*]

[1] Beijing Institute of Dental Research, Beijing Stomatological Hospital, Capital Medical University, Beijing, China

[*]**Corresponding authors. Email Addresses:**

E-mail: shuaiyu369@163.com

E-mail: 2265804732@qq.com

Supplemental figure 1. CRISPR/Cas9 genome editing of mouse *Ano5*.

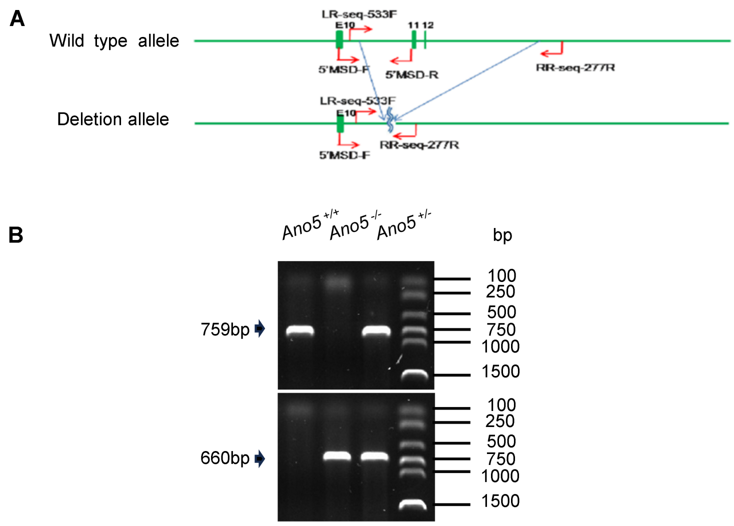

(A) Schematic diagram of the *Ano5* knockout design.

(B) Genotype identification of mice by genomic PCR.

Supplemental figure 2. Gene Ontology analysis based on RNA sequencing.

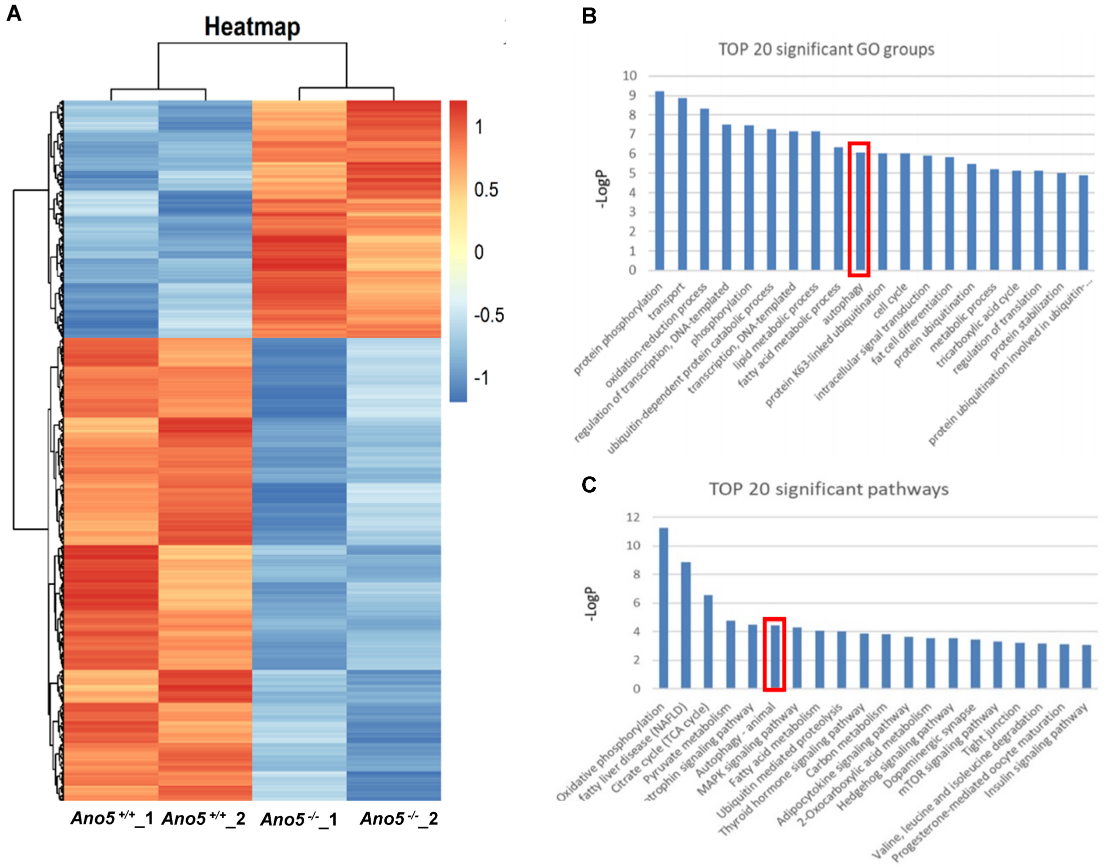

(A) Heatmap of differentially expressed genes in mCOBs from *Ano5*$^{+/+}$ and *Ano5*$^{-/-}$ groups.

(B) Top 20 clusters of GO enrichment analysis.

(C) Top 20 pathways of GO enrichment analysis.

Supplemental figure 3. The availability of lysosomes increased in the absence of *Ano5*.

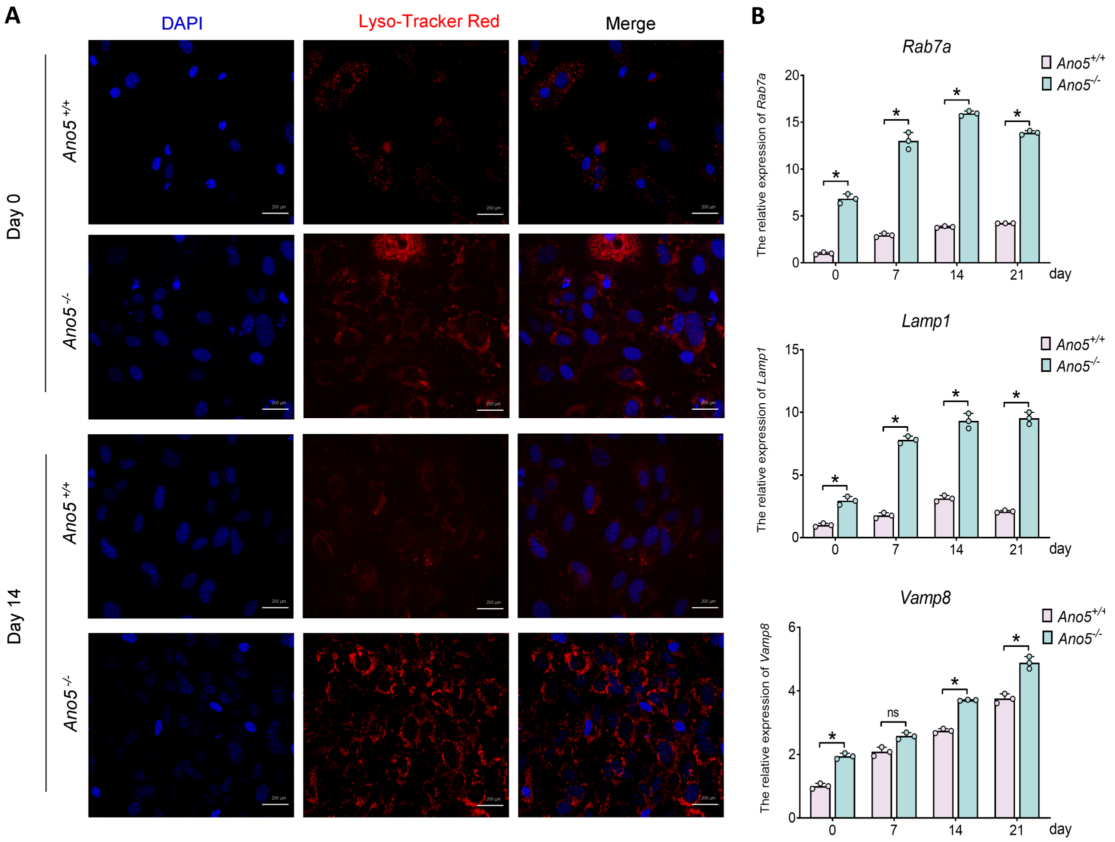

(A) Lysosomal probe analysis of lysosomal content in mCOBs from *Ano5*$^{+/+}$ and *Ano5*$^{-/-}$ groups during osteogenic differentiation. (B) qRT-PCR analysis of *Rab7a*, *Lamp1*, and *Vamp8* expression in mCOBs from *Ano5*$^{+/+}$ and *Ano5*$^{-/-}$ groups during osteogenic differentiation. Data are represented as mean±SD. **P* < 0.05; ns, *P* > 0.05, as assessed by 1-way ANOVA followed by Tukey's post-hoc test.

Supplemental figure 4. The aberrant osteogenic and autophagic activities in the bones of *Ano5*$^{-/-}$ mice.

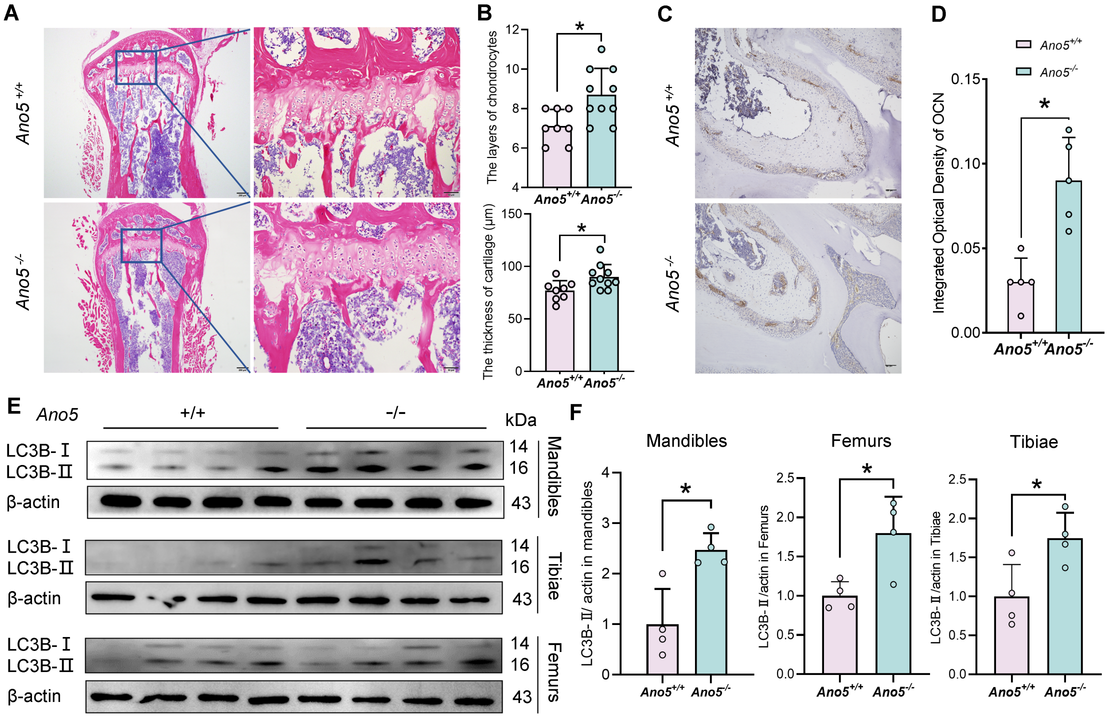

(A) Cartilage of the tibiae in *Ano5*$^{+/+}$ and *Ano5*$^{-/-}$ mice. (B) Chondrocyte layers and cartilage thickness as shown in A. (C) Immunohistochemical staining revealed the expression of OCN around the alveolar ridge. (D) Quantification of integrated optical density of OCN in C. (E) Western blot analysis of LC3B-II expression in mandibles, tibiae, and femurs of *Ano5*$^{+/+}$ and *Ano5*$^{-/-}$ mice. (F) Quantification of LC3B-II/β-actin ratios in E. Data are represented as mean±SD. *$P < 0.05$; ns, $P > 0.05$, as assessed by 1-way ANOVA followed by Tukey's post-hoc test.

Supplemental figure 5. The inhibition of ULK1 had no effect on the osteogenic capacity of *Ano5*$^{-/-}$ mCOBs.

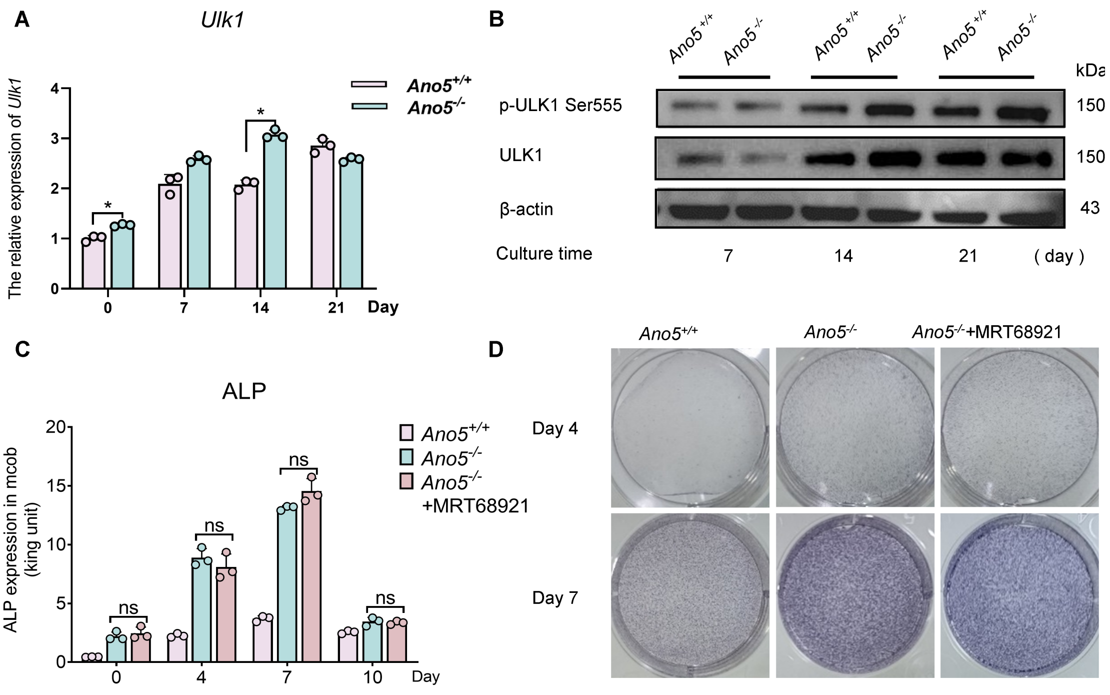

(A) qRT-PCR analysis of *Ulk1* expression in mCOBs from *Ano5*$^{+/+}$ and *Ano5*$^{-/-}$ groups during osteogenic differentiation. (B) Western blot analysis of p-ULK1 Ser 555 and ULK1 protein levels in mCOBs from *Ano5*$^{+/+}$ and *Ano5*$^{-/-}$ groups during osteogenic differentiation. (C) ALP activity assay measurement of mCOBs following treatment with MRT68921. (D) ALP staining of mCOBs following treatment with MRT68921 (a ULK1 inhibitor). Data are represented as mean±SD. *P < 0.05; ns, P ＞ 0.05, as assessed by 1-way ANOVA followed by Tukey's post-hoc test.

Supplemental figure 6. The p-AKT expression was upregulated in the absence of *Ano5*.

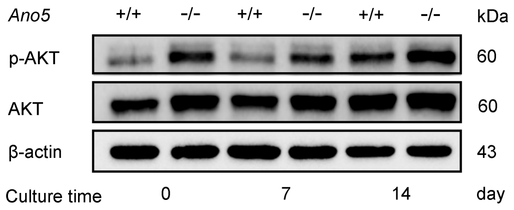

Western blot analysis of p-AKT and AKT protein levels in mCOBs from *Ano5*$^{+/+}$ and *Ano5*$^{-/-}$ groups during osteogenic differentiation.

Supplemental figure 7. Cortical bone of femurs and tibiae after treatment with 3MA.

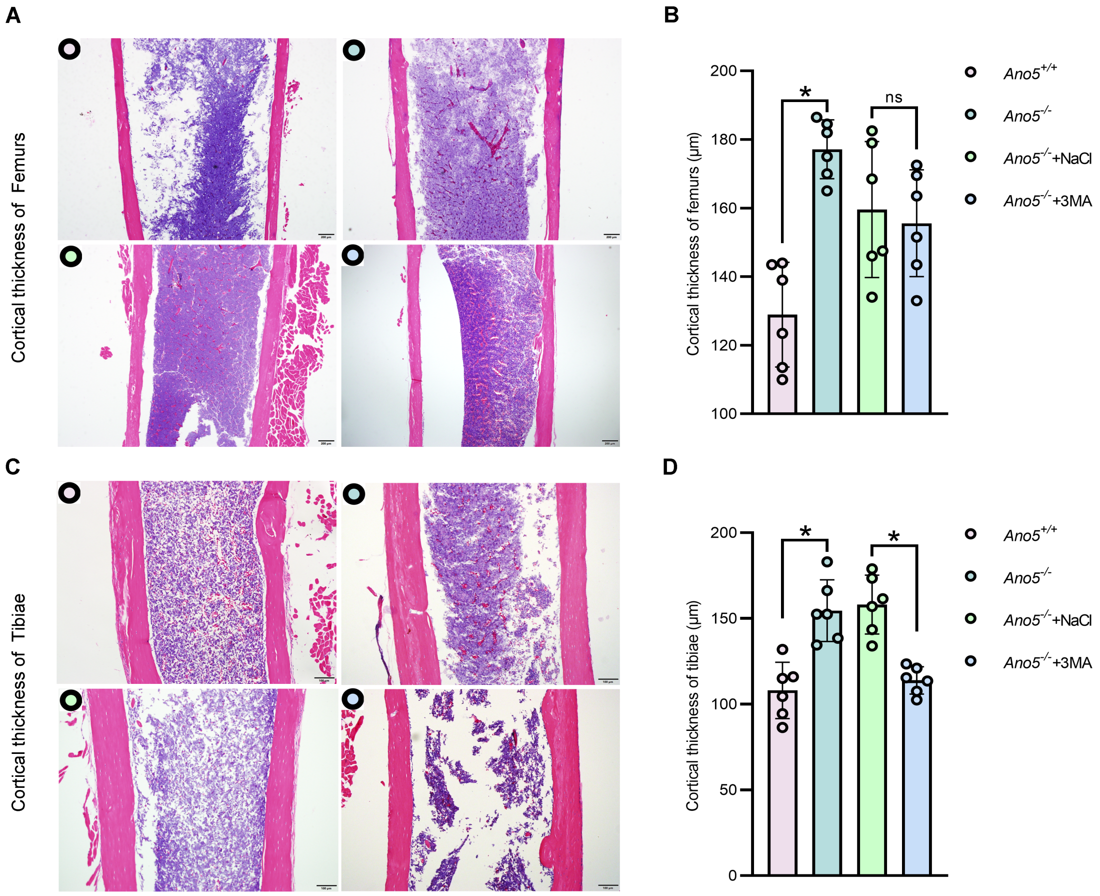

(A) Cortical bone of the femurs (n=6 pre group). (B) Quantification of femoral cortical bone thickness in A. (C) Cortical bone of the tibiae (n=6 pre group). (D) Quantification of tibial cortical bone thickness in D. Data are represented as mean±SD. *$P < 0.05$; ns, $P > 0.05$, as assessed by 1-way ANOVA followed by Tukey's post-hoc test.

Supplemental figure 8. The expression of ATG9A in tibiae.

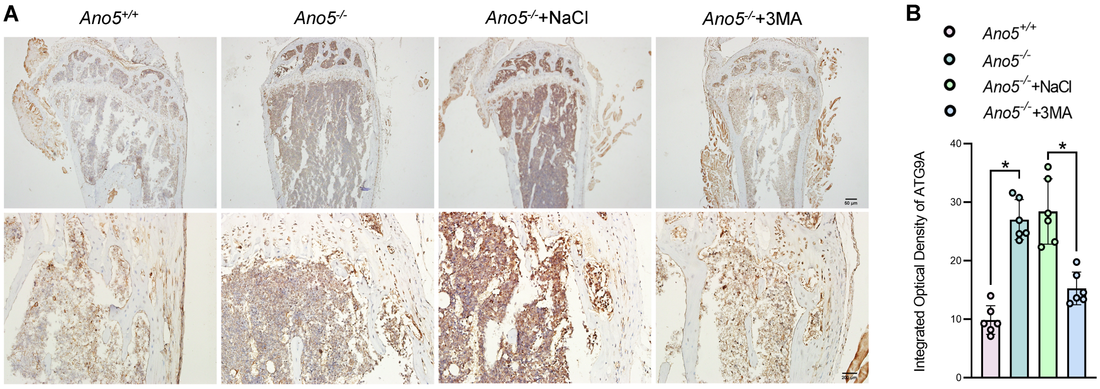

(A) Immunohistochemical staining revealed the expression of ATG9A in tibiae. (n=6 pre group). (B) Quantification of integrated optical density of ATG9A in A. Data are represented as mean±SD. *P < 0.05; ns, P > 0.05, as assessed by 1-way ANOVA followed by Tukey's post-hoc test.

Supplementary Table.1 List of antibodies used in this manuscript

Antibody	Company	Cat.#
LC3B (D11) XP® Rabbit mAb	Cell Signaling Technology	3868
Akt (pan) (C67E7) Rabbit mAb	Cell Signaling Technology	4691
Phospho-Akt (Ser473) (D9E) XP® Rabbit mAb	Cell Signaling Technology	4060
Phospho-AMPKα1 (Ser485) (45F5) Rabbit mAb	Cell Signaling Technology	2537
Phospho-ULK1 (Ser555) (D1H4) Rabbit mAb	Cell Signaling Technology	5869
Anti-AMPK alpha 1 antibody [Y365]	abcam	ab32047
β-Actin Rabbit mAb (High Dilution)	ABclonal	Ac026
SQSTM1/p62 Rabbit mAb	ABclonal	A19700
Beclin 1 Rabbit pAb	ABclonal	A7353
Collagen I / COL1A1 Rabbit pAb	ABclonal	A1352
Cy3-conjugated Goat anti-Rabbit IgG (H+L)	ABclonal	AS007
Osteocalcin Antibody	Affinity Biosciences	DF12303
Anti-ATG9A Antibody	BOSTER	A03757-3
Anti-ATG9A Antibody	BOSTER	A03757-2
ULK1 Rabbit pAb	Zen-Bio	381887

Supplementary Table.2 The primers used in this manuscript

	**Forward Primer**	**Reverse Primer**
Atg3	ACACGGTGAAGGGAAAGGC	TGGTGGACTAAGTGATCTCCAG
Atg4b	AGTTCCTTGGCTGTTCACATAG	CGATGGCCTGTTGGTGACTT
Atg5	TGTGCTTCGAGATGTGTGGTT	ACCAACGTCAAATAGCTGACTC
Becn1	CAGCCTCTGAAACTGGACACGA	CTCTCCTGAGTTAGCCTCTTCC
Atg7	GTTCGCCCCCTTTAATAGTGC	TGAACTCCAACGTCAAGCGG
Atg9a	ATGGCTCTCTTATCACCATCCT	TGGATCTCCCAATAGCAGCAA
Ulk1	AAGTTCGAGTTCTCTCGCAAG	CGATGTTTTCGTGCTTTAGTTCC
Actb	GTGACGTTGACATCCGTAAAGA	GCCGGACTCATCGTACTCC
Rab7a	AGGCTTGGTGCTACAGGAAAA	CTTGGCCCGGTCATTCTTGT
Vamp8	AGTGGGAGTGCCGGAAATG	TGAAGTGTTCAGACGTGGCTT
Lamp1	CAGCACTCTTTGAGGTGAAAAAC	ACGATCTGAGAACCATTCGCA
Ocn	GAACAGACAAGTCCCACACAGC	TCAGCAGAGTGAGCAGAAAGAT
Col1a1	TTCTCCTGGTAAAGATGGTGC	GGACCAGCATCACCTTTAACA
