# Supplementary material for: Anoctamin-5 deficiency enhances ATG9A-dependent autophagy, inducing osteogenesis and gnathodiaphyseal dysplasia–like bone formation
Source: JCI Insight. 2025 Mar 11;10(8):e189817. doi: 10.1172/jci.insight.189817 (PMC12016930; doi:10.1172/jci.insight.189817)

Full unedited gel for Figure 1A

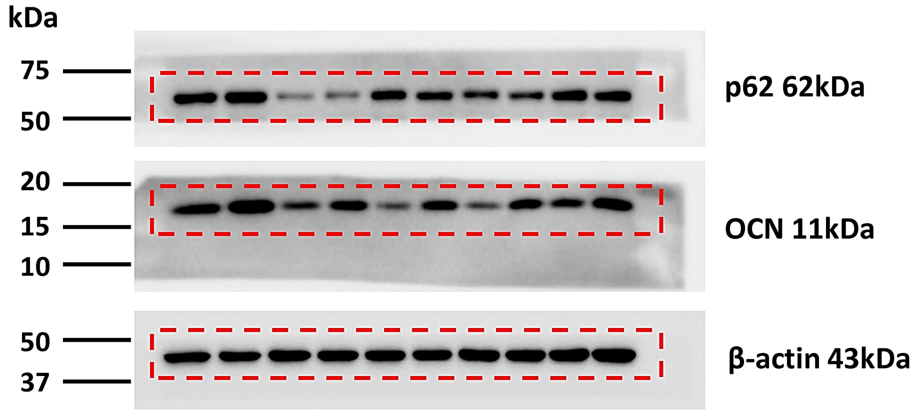

Full unedited gel for Figure 1B

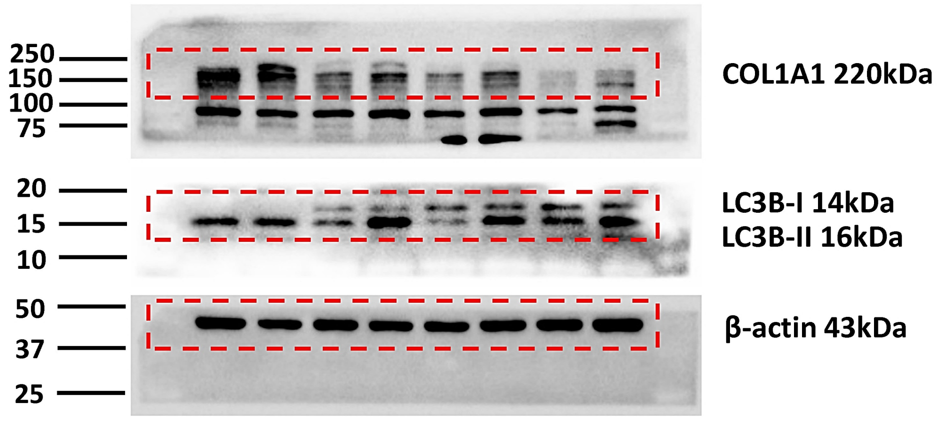

Full unedited gel for Figure 1C

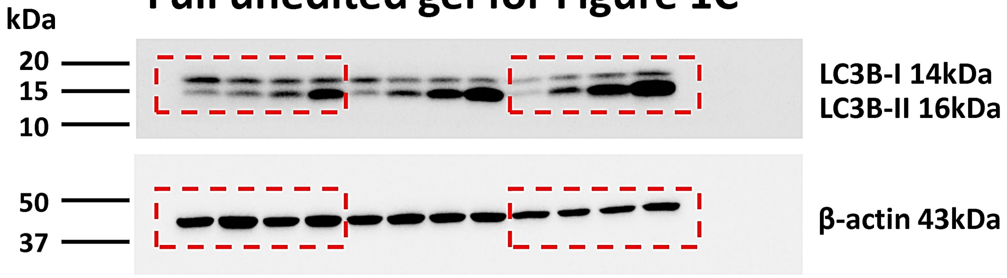

Full unedited gel for Figure 2A

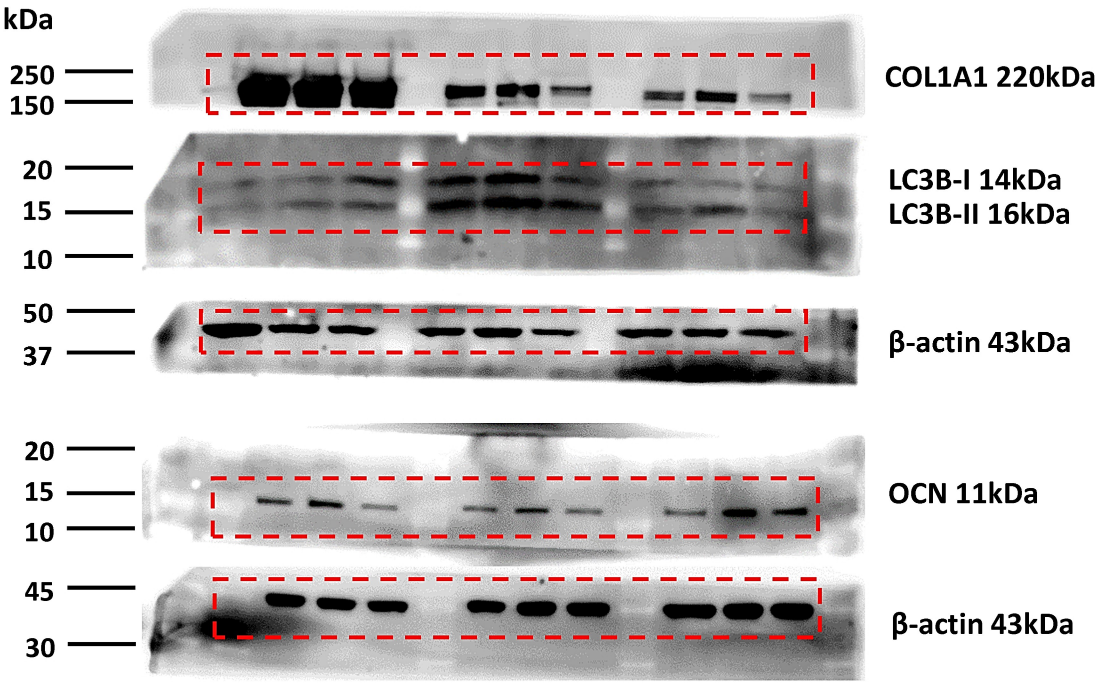

Full unedited gel for Figure 3B

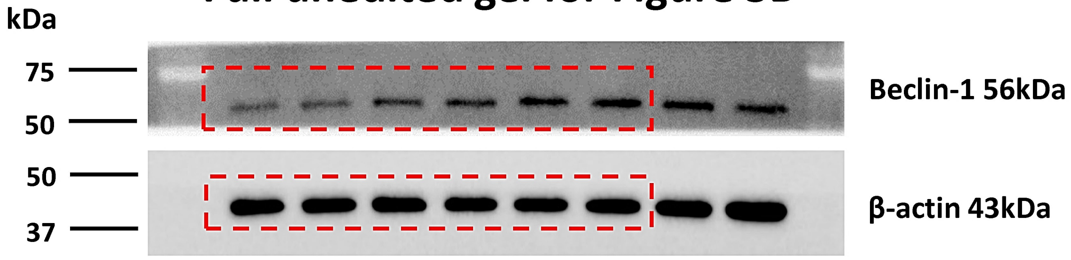

Full unedited gel for Figure 3C & Figure S6

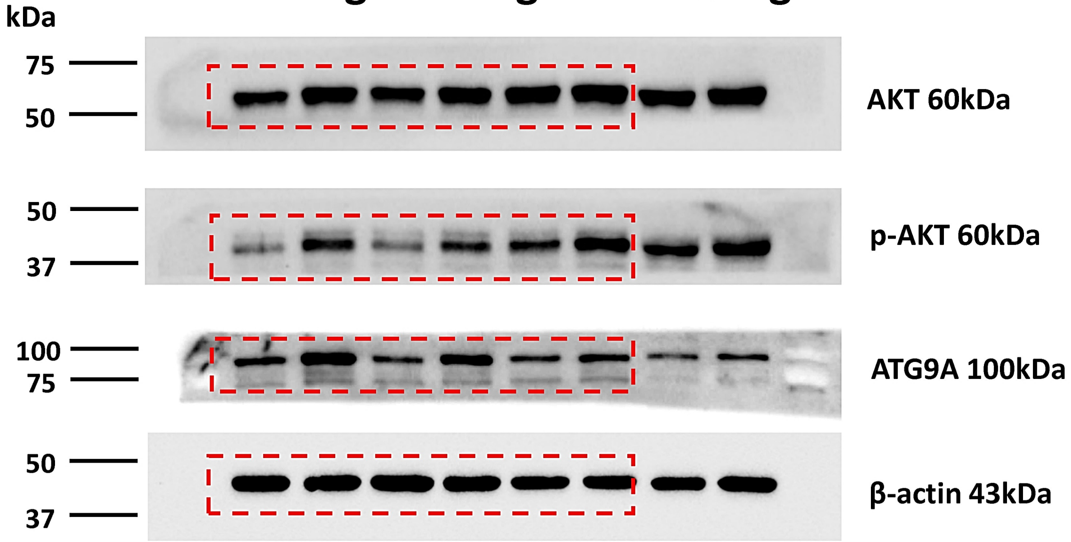

Full unedited gel for Figure 4A

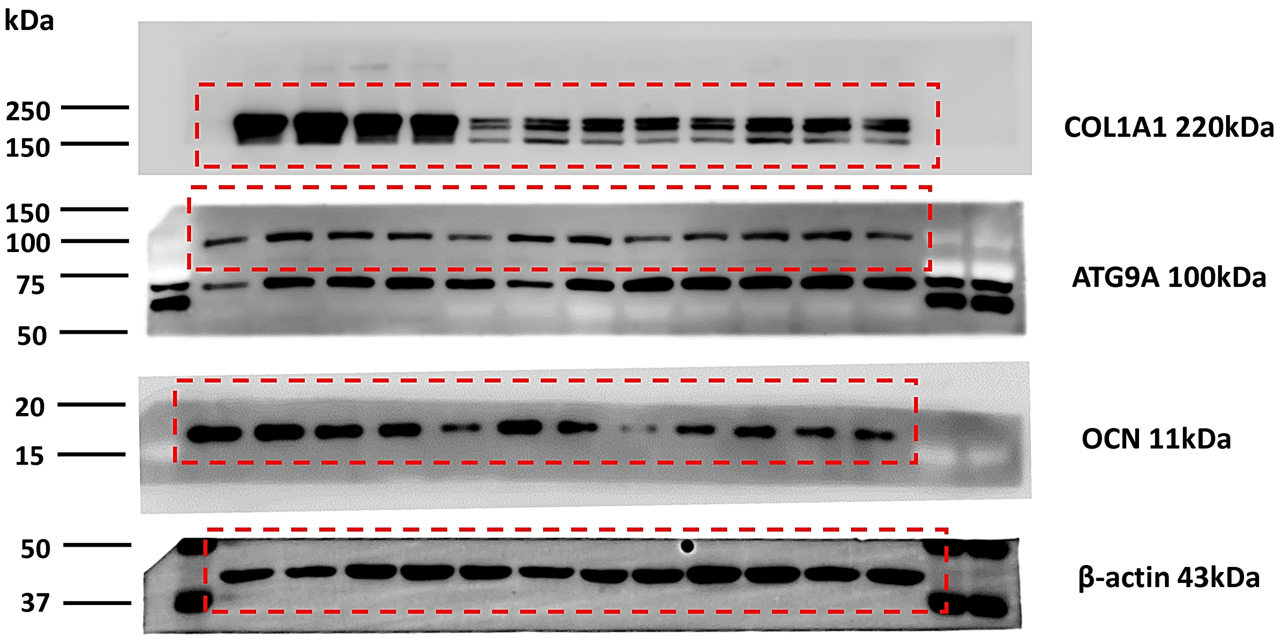

Full unedited gel for Figure 5B

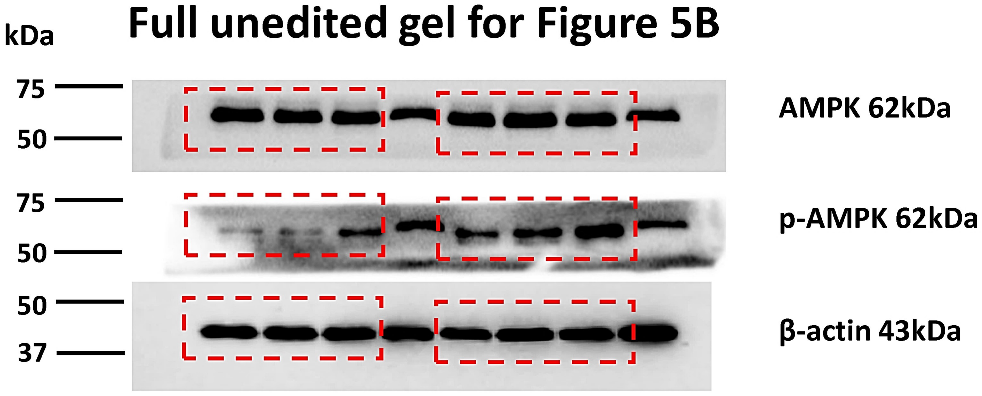

Full unedited gel for Figure 6A

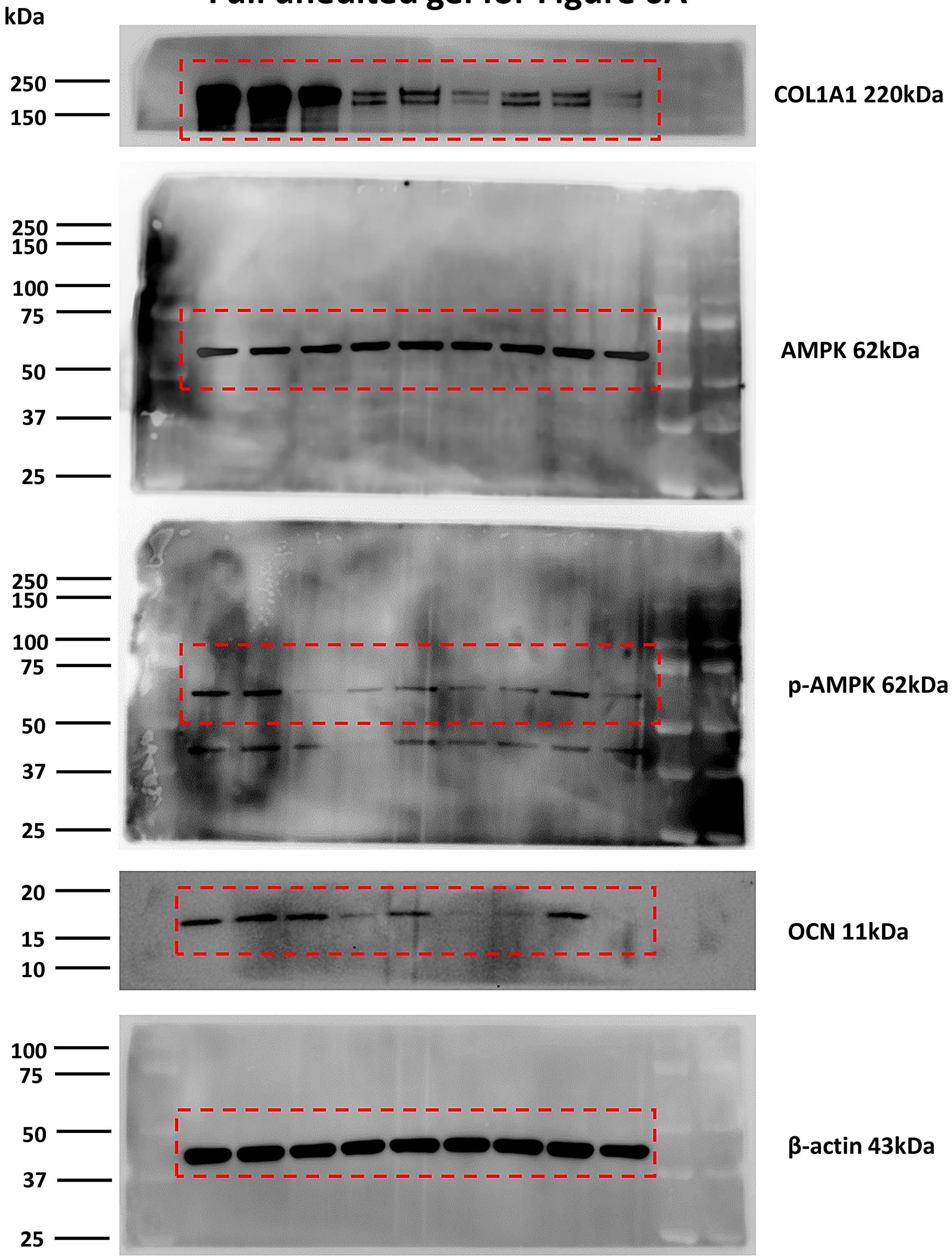

Full unedited gel for Figure 6F

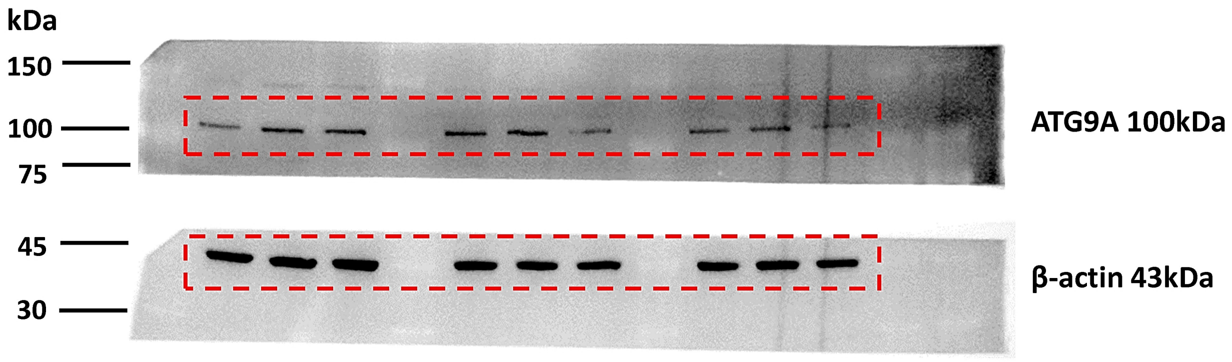

Full unedited gel for Figure 6G

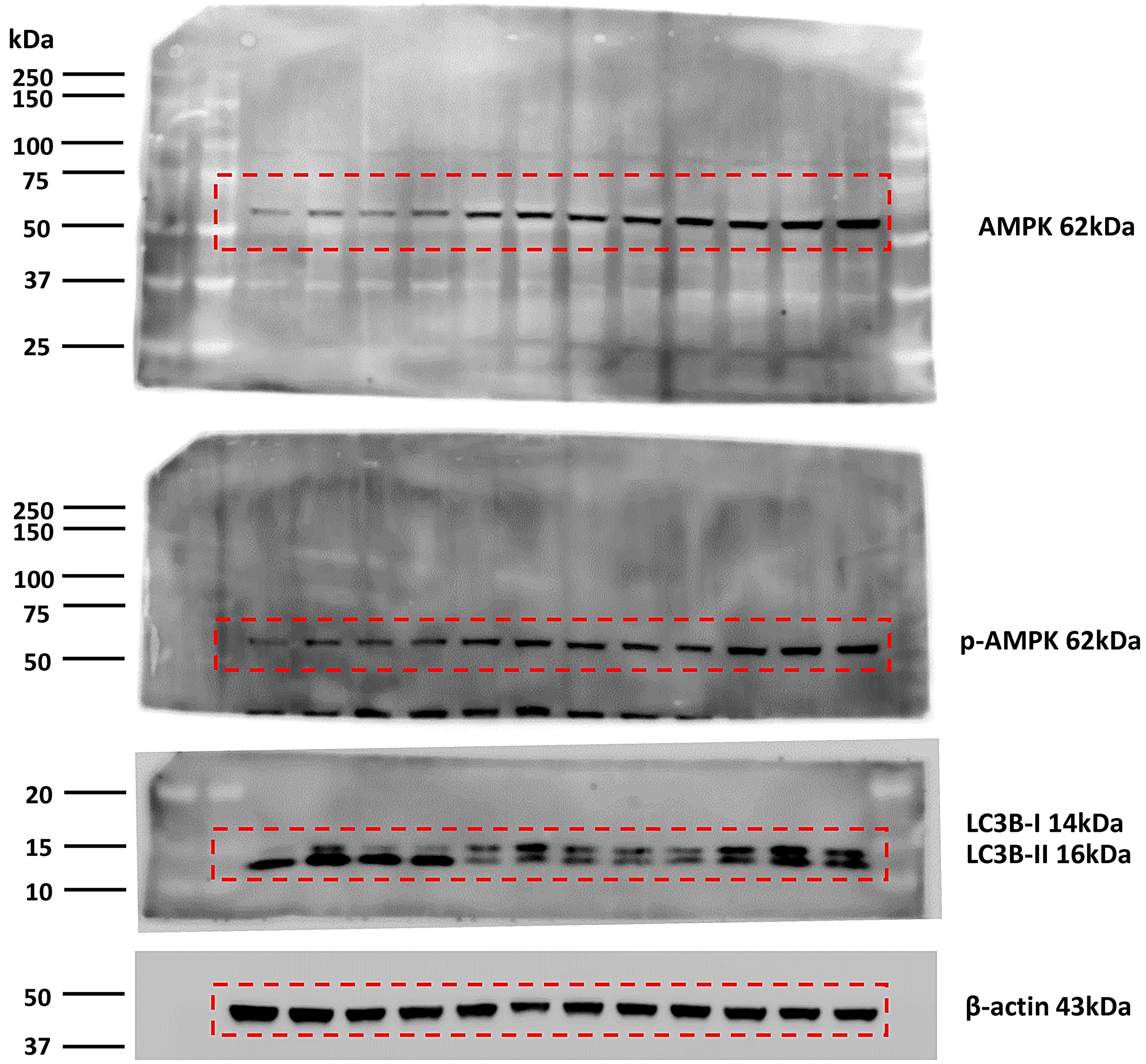

Full unedited gel for Figure 7B

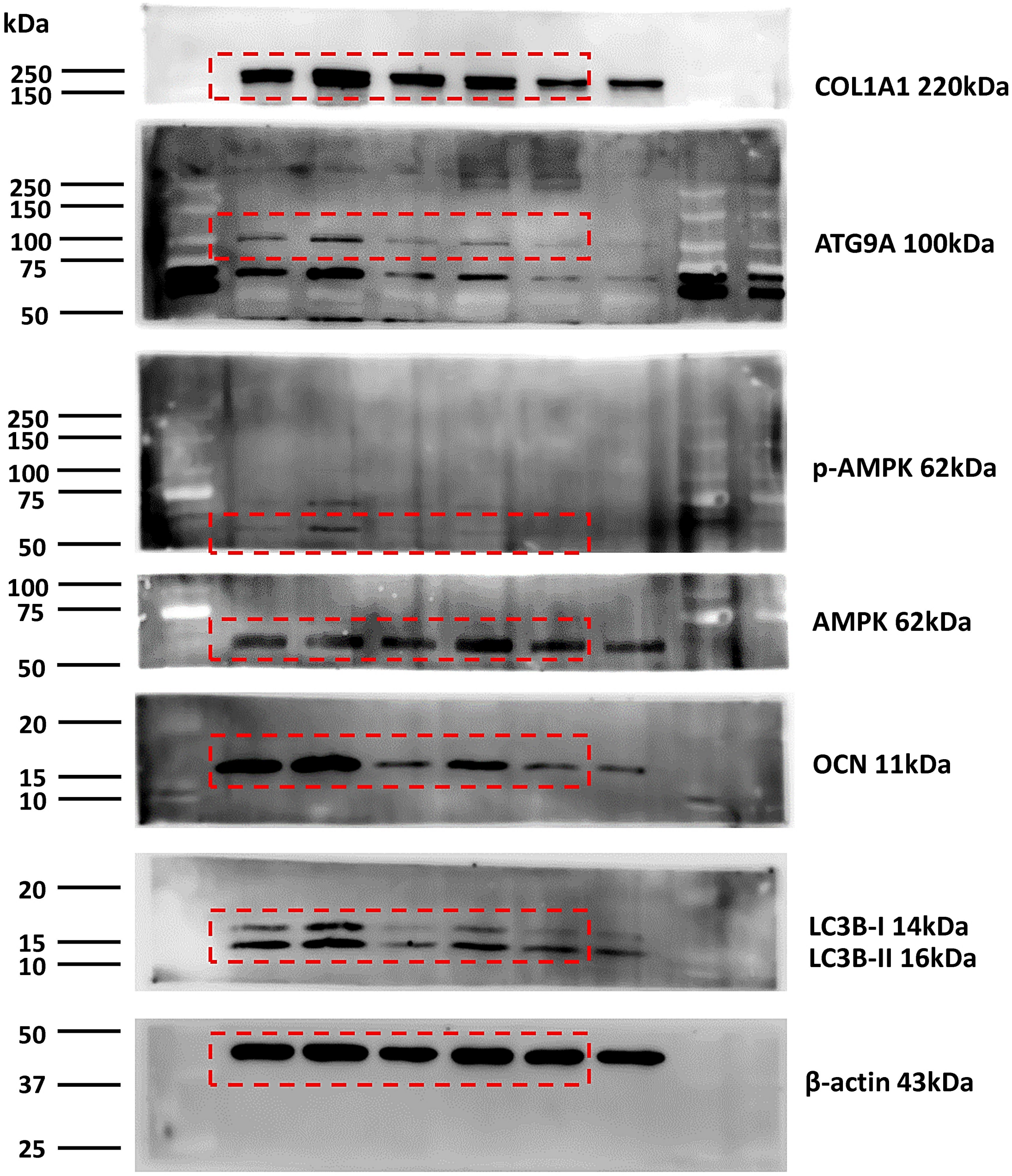

Full unedited gel for Figure 9L

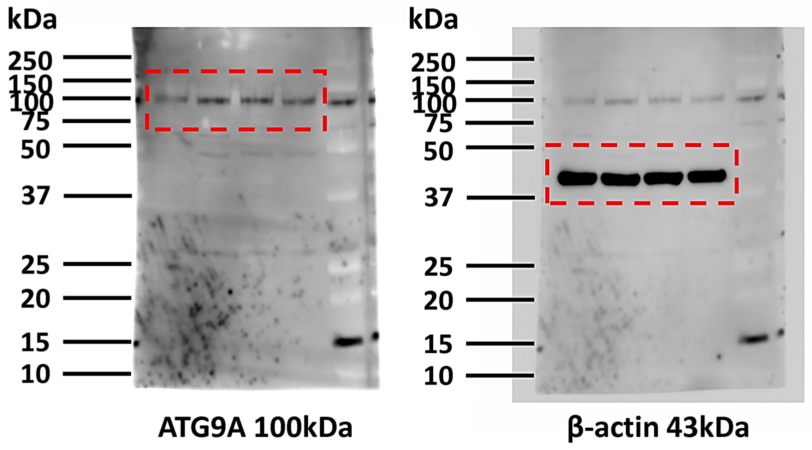

Full unedited gel for Figure S4E

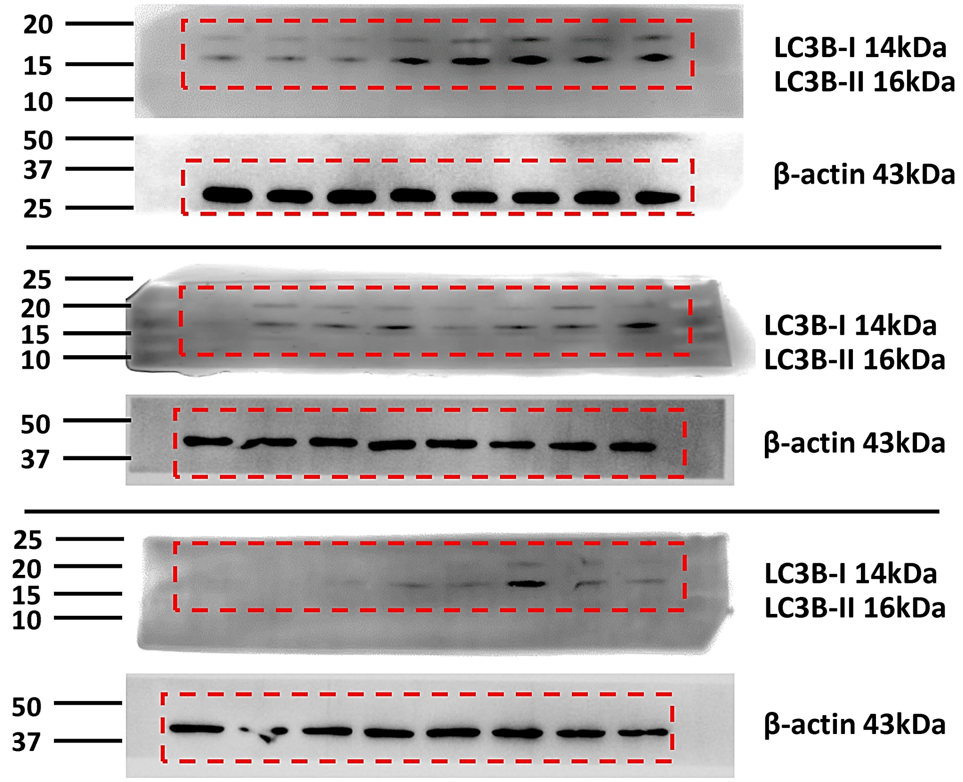

Full unedited gel for Figure S5B

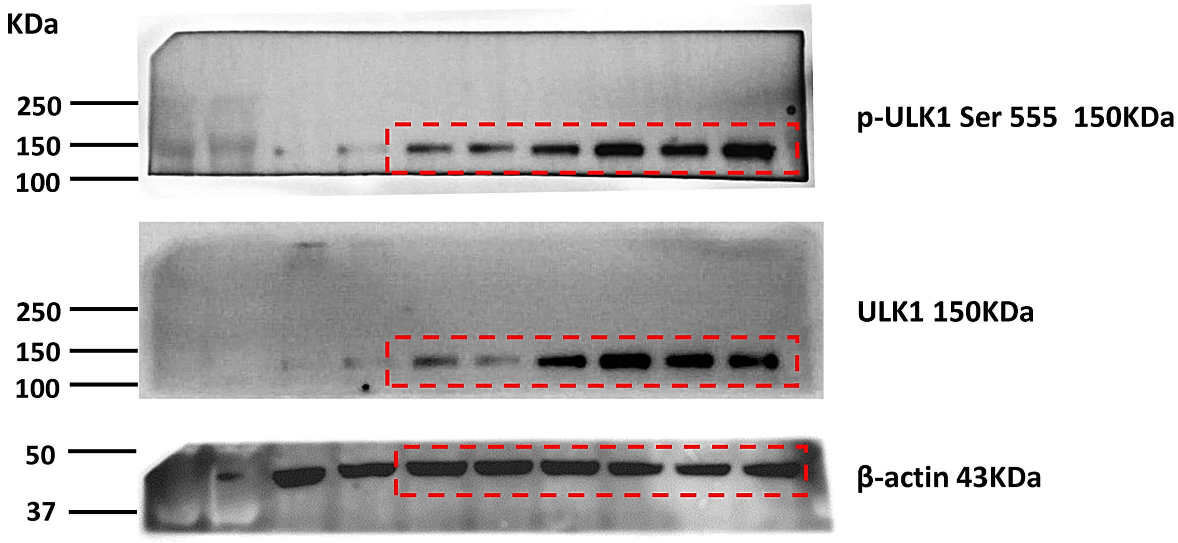

Supplement: Unedited blot and gel images [file jciinsight-10-189817-s159.pdf]
